# Antioxidant, Antigenotoxic and Cytotoxic Activity of Essential Oils and Methanol Extracts of *Hyssopus officinalis* L. Subsp. *aristatus* (Godr.) Nyman (Lamiaceae)

**DOI:** 10.3390/plants10040711

**Published:** 2021-04-07

**Authors:** Tijana Mićović, Dijana Topalović, Lada Živković, Biljana Spremo-Potparević, Vladimir Jakovljević, Sanja Matić, Suzana Popović, Dejan Baskić, Danijela Stešević, Stevan Samardžić, Danilo Stojanović, Zoran Maksimović

**Affiliations:** 1 Institute for Medicines and Medical Devices of Montenegro, Bulevar Ivana Crnojevića 64a, 81000 Podgorica, Montenegro; tijana.micovic@calims.me; 2 Department of Pathobiology, Faculty of Pharmacy, University of Belgrade, Vojvode Stepe 450, 11000 Belgrade, Serbia; dijana.topalovic@pharmacy.bg.ac.rs (D.T.); lada.zivkovic@pharmacy.bg.ac.rs (L.Ž.); biljana.potparevic@pharmacy.bg.ac.rs (B.S.-P.); 3 Department of Physiology, Faculty of Medical Sciences, University of Kragujevac, Svetozara Markovića 69, 34000 Kragujevac, Serbia; drvladakgbg@yahoo.com; 4 Department of Human Pathology, First Moscow State Medical University I. M. Sechenov, Trubetskaya Street 8, Str. 2, 119991 Moscow, Russia; 5 Department of Pharmacy, Faculty of Medical Sciences, University of Kragujevac, Svetozara Markovića 69, 34000 Kragujevac, Serbia; sanjad.matic@gmail.com; 6 Department of Microbiology and Immunology, Center for Molecular Medicine and Stem Cell Research, Faculty of Medical Sciences, University of Kragujevac, Svetozara Markovića 69, 34000 Kragujevac, Serbia; popovic007@yahoo.com (S.P.); dejan.baskic@gmail.com (D.B.); 7 Public Health Institute, Nikole Pašića 1, 34000 Kragujevac, Serbia; 8 Faculty of Natural Sciences and Mathematics, University of Montenegro, Džordža Vašingtona bb, 81000 Podgorica, Montenegro; danijela.stesevic@ucg.ac.me; 9 Department of Pharmacognosy, Faculty of Pharmacy, University of Belgrade, Vojvode Stepe 450, 11000 Belgrade, Serbia; stevan.samardzic@pharmacy.bg.ac.rs; 10 Department of Botany, Faculty of Pharmacy, University of Belgrade, Vojvode Stepe 450, 11000 Belgrade, Serbia; dancho@pharmacy.bg.ac.rs

**Keywords:** *Hyssopus officinalis*, antioxidant activity, antigenotoxic activity, comet assay, cytotoxic activity, HeLa cell line, essential oil, methanol extract, GC-MS, LC-DAD-MS

## Abstract

*Hyssopus officinalis* L. is a well-known aromatic plant used in traditional medicine and the food and cosmetics industry. The aim of this study is to assess the antioxidant, genotoxic, antigenotoxic and cytotoxic properties of characterized hyssop essential oils and methanol extracts. Chemical composition was analyzed by gas chromatography - mass spectrometry (GC-MS) and liquid chromatography with diode array detection and mass spectrometry (LC-DAD-MS), respectively. Antioxidant activity was examined by 2,2-diphenyl-1-picrylhydrazyl (DPPH) and ferric reducing/antioxidant power (FRAP) tests; genotoxic and antigenotoxic activity were examined by the comet assay, while cytotoxicity was evaluated by the 3-(4,5-dimethylthiazol-2-yl)-2,5 diphenyltetrazolium bromide dye (MTT) test against tumor cell lines (SW480, MDA-MB 231, HeLa) and non-transformed human lung fibroblast cell lines (MRC-5). The essential oils were rich in monoterpene hydrocarbons (e.g., limonene; 7.99–23.81%), oxygenated monoterpenes (1,8-cineole; 38.19–67.1%) and phenylpropanoids (methyl eugenol; 0.00–28.33%). In methanol extracts, the most abundant phenolics were chlorogenic and rosmarinic acid (23.35–33.46 and 3.53–17.98 mg/g, respectively). Methanol extracts expressed moderate to weak antioxidant activity (DPPH IC_50_ = 56.04–199.89 µg/mL, FRAP = 0.667–0.959 mmol Fe^2+^/g). Hyssop preparations significantly reduced DNA damage in human whole blood cells, induced by pretreatment with hydrogen peroxide. Methanol extracts exhibited selective and potent dose- and time-dependent activity against the HeLa cell line. Results of the current study demonstrated notable *H*. *officinalis* medicinal potential, which calls for further investigation.

## 1. Introduction

Hyssop, *Hyssopus officinalis* L. (Lamiaceae), is a shrubby perennial herbaceous plant, distributed mostly in the Mediterranean area [1,2,3]. In Montenegro and Serbia, *Hyssopus officinalis* L. subsp. *aristatus* (Godr.) Nyman (syn. *H. officinalis* L. subsp. *pilifer* (Gris. ex Pant.) Murb.) can be found in plant communities of rocky pastures [2].

Hyssop herb (*Hyssopi herba*) and its pharmaceutical preparations (infusions, syrups, tinctures, extracts) have been used in traditional medicine since ancient times as antiseptic, carminative, diaphoretic, emenagogue, expectorant, muscle relaxant, stomachic and tonic agents. As an aromatic plant, it is also used in the food and cosmetics industry [4,5,6].

Essential oil is the most important and the most frequently investigated product of hyssop. Available literature data on wild and cultivated plants indicate that its herb yields 0.3–1% of essential oil with isopinocamphone as the dominant compound, along with pinocamphone, *β*-pinene, 1,8-cineole, pinocarvone, linalool, sabinene and methyl eugenol [7,8,9]. Beside the essential oil, hyssop herb contains flavonoids and phenolic acids, tannins, diterpene lactones (marrubiin) and triterpenoid compounds such as ursolic and oleanolic acid [5,7,10].

Antimicrobial activity is one of the most commonly examined pharmacological effects of various hyssop preparations. Extensive experimental evidence also speak in favor of antioxidant [10], antiviral [11], sedative and anxiolytic [12,13], spasmolytic [14], anti-inflammatory [15], antiulcer [16], anti-asthmatic [17] and antidiabetic activities of the hyssop herb [18]. However, despite the diversity in scientific information on pharmacological activities of *Hyssopi herba* and its preparations, genotoxicity, antigenotoxicity and cytotoxic activity of the essential oils and polar extracts of this herbal drug are still insufficiently investigated.

In attempt to better understand medicinal potentials of *H. officinalis* herb in this field, we designed and performed a set of chemical and physiological investigations on the plant material collected from five wild-growing populations in Montenegro and on one commercial sample available in herbal apothecaries in Serbia, and manufactured by a local enterprise from wild-growing sources.

*Hyssopi herba,* as an herbal medicinal substance and a raw material for pharmaceutical and related industries, is neither official in pharmacopoeias, nor listed in the other well-established documents. Therefore, the question of its pharmaceutical quality still remains open. Having in mind that growing (for plants harvested from the wild) and/or cultivation conditions can significantly affect the composition and activity of a given plant, it would explain the need for sampling at different sites, as well as the use of a commercial sample-that is something that a person can find, if needed.

Consequently, the main objectives of the present work were to quantify the levels of in vitro antioxidant activity by commonly used 2,2-diphenyl-1-picrylhydrazyl (DPPH) and ferric reducing/antioxidant power (FRAP) tests, to assess potential genotoxicity/antigenotoxicity by comet assay, and to determine the overall cytotoxic, cytostatic and cytocidal effects against human tumor and non-transformed human lung fibroblast cell lines of investigated hyssop essential oils and/or extracts, with respect to their chemical composition.

## 2. Results and Discussion

### 2.1. Essential Oils Compositions

Produced oils were pale yellowish-green liquids with characteristic pleasant odors. The yields ranged from 0.4% to 0.79% (*v/w*) for samples collected from the wild in Montenegro. However, the highest yield was obtained from the commercial sample (Serbia), amounting to 1% (*v/w*). The results of the gas chromatography coupled with mass spectrometry (GC-MS) analysis of the essential oils (1EO–6EO) obtained from tested samples of *H*. *officinalis* subsp. *aristatus* (1–6, Table 1) are presented in Table 2. Overall, 12 to 16 compounds were identified depending on the sample, which is more than 98% of the total oil on average, with one exception (sample 2EO), where the percentage of identified compounds was lower (86.84%).

The dominant group of identified volatiles was monoterpenes (70.88–95.91%). Their oxygenated derivatives were the most abundant (41.7–68.8%), among which 1,8-cineole and *cis*-pinocamphone were found in the highest contents. Only in the sample 4EO, the content of monoterpene hydrocarbons (43.53%) was higher than the content of oxygenated monoterpene derivatives. With regard to the monoterpene hydrocarbons, the dominant constituents were *β*-pinene and limonene. Methyl eugenol (up to 28.33%), the only compound belonging to the phenylpropanoid group, and sesquiterpene hydrocarbons (up to 0.87%) were present in a lower percentage. Monoterpene hydrocarbons, such as *β*-pinene, limonene, *Z*-*β*-ocimene, *α*-pinene, sabinene and the oxygenated monoterpene derivative myrtenal, were present in all the investigated samples.

**Figure 1:**
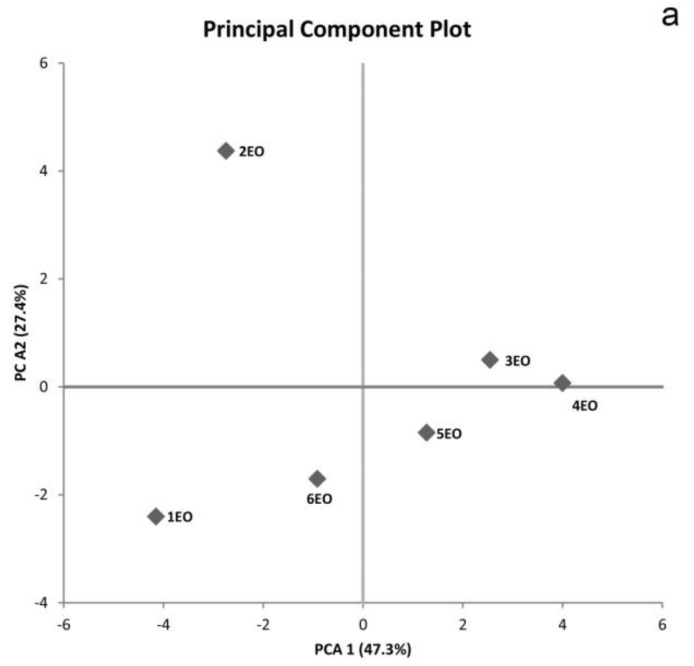

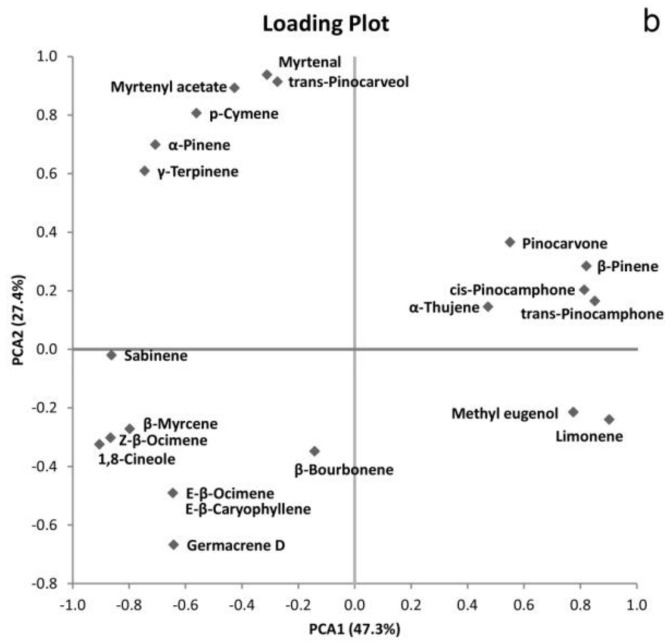
Plots of principal component analysis (PCA) scores (**a**) and loadings (**b**) along the first two principal components (PCs) extracted from the dataset of *Hyssopus officinalis* essential oils from six mutually independent sources, as listed in Table 2.

The principal component analysis (PCA) confirmed the existence of significant chemical variation in the investigated essential oils. Performed on the entire dataset, PCA detected five principal components (PCs), with the first three accounting together for more than 86% of total variance (Figure 1). The constituents of the oils which contribute the most to the corresponding PCs are listed in Table S1 (Supplementary Material), along with their loadings and scores. Along the first PC axis, the highest number of significant characters of separation (factor loadings higher than ±0.7) was detected. The second and third PCs further underscored the chemical variations between samples.

**Figure 2:**
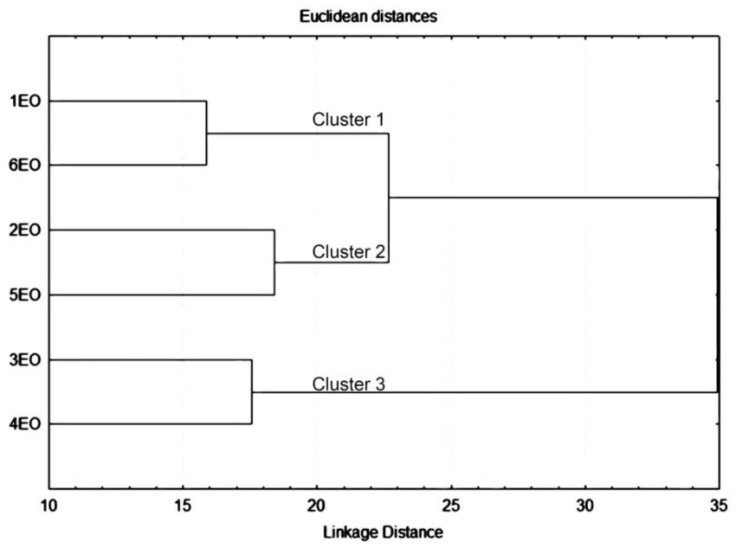
Dendrogram representing the similarity relations of the chemical composition of *Hyssopus officinalis* essential oils. For this analysis, an entire chromatographic dataset was taken into consideration. Amalgamation rule: single-linkage. Distance metric is Euclidean distances (non-standardized).

The cluster analysis of entire dataset revealed the similarity in the composition of essential oils from the commercial sample of *H. officinalis* and plants collected from the locality Cuce in Montenegro (Cluster 1), plants collected from localities Kuči and Piperi (Cluster 2) and plants collected from localities Šavnik and Piva (Cluster 3), as shown in Figure 2. The results indicated that the classification proposed by the PCA and hierarchical cluster analysis (HCA) are in good agreement.

Considering the previously reported literature data, numerous compounds have formerly been identified in essential oils of hyssop and several chromatographic profiles have been described.

Differences in oil composition (deriving from climatic conditions, the origin of plant material, subspecies or variety, developmental stages, soil type, cultivation technologies, extraction methods, etc.) determine its organoleptic and physiological properties, and hence, its possibilities of application [1,5,7,19,20,21,22,23].

The most characteristic and important components of so far investigated *H. officinalis* essential oils are 1,8-cineole [1,21,22], *cis*-pinocamphone, *trans*-pinocamphone and their precursor *β*-pinene [1,19]. Among the other principal constituents, pinocarvone, sabinene, germacrene D, germacren D-4-ol, *α*-, *β*-phellandrene, 4-carvomenthenol, thymol, carvacrol, elemol, limonene, linalool, *α*-terpinene, myrtenol, myrtenyl acetate and methyl eugenol were also reported [7].

With regard to the hyssop growing in Serbia, Mitić et al. (2000) identified *cis*-pinocamphone (44.7%) as the most abundant constituent of its essential oil, followed by *trans*-pinocamphone (14.1%), germacren-D-11-ol (5.7%) and elemol (5.6%) [19]. Gorunović et al. (1995) examined hyssop from the territory of Montenegro. The main constituents were methyl eugenol (38.30%), limonene (37.40%) and *β*-pinene (9.6%) [20].

Hajdari A. et al. (2018) investigated the composition of the essential oil of wild-growing *H. officinalis* subsp. *aristatus* (aerial parts) from five different localities in Kosovo, and found that in four out of five samples, the dominant compound was *cis*-pinocamphone, with the content ranging between 30.44% and 57.73%. In a sample from one of the localities, the dominant compound was 1,8-cineole (45.27%). The same authors found that the content of *trans*-pinocamphone (14.76%) was significant in one of the samples, as well as that of *β*-pinene (23.31%) and caryophyllene oxide (12.66%) [21].

The essential oils obtained from wild-growing *H. officinalis* L. subsp. *aristatus* in Bulgaria in two stages of development (during the flower bud formation and in the full bloom) were similar in composition, with 1,8-cineole (48.2% and 39.6%), isopinocamphone (16.3% and 29.2%) and *β*-pinene (11.4% and 39.6%) as the major constituents. The essential oil obtained from cultivated *H. officinalis* contained larger amounts of isopinocamphone (40.2%), pinocamphone (10.3%) and *β*-pinene (14.2%), but no traces of 1,8-cineole [22].

In the essential oil of wild-growing *H. officinalis* subsp. *aristatus* (aerial parts) native to Italy, the main compound was linalool (35.3–51.2%), whereas methyl eugenol (7.3–22.7%), limonene (3.7–4.4%), germacrene D (1.9–4.1%), (*Z*)-*β*-ocimene (5.1–5.8%) and (*E*)-*β*-ocimene (2.1–5.3%) were reported as well [5].

Our results revealed three chromatographic profiles in investigated essential oils of wild-growing plants from Montenegro. The essential oil obtained from plants collected from the locality Cuce in Montenegro (sample 6EO) was similar with the essential oil of the commercial sample from southeastern Serbia (sample 1EO), being high in 1,8-cineole and relatively rich in *β*-pinene, but low in *cis*-pinocamphone. On the other hand, the essential oils of plants collected from the localities Šavnik and Piva in Montenegro (samples 3EO and 4EO, respectively) stood out for being high in *β*-pinene, limonene, *cis*-pinocamphone and methyl eugenol, but relatively low in 1,8-cineol at the same time. Finally, the essential oils obtained from the plants collected from the localities Kuči and Piperi in Montenegro (samples 2EO and 5EO, respectively) appeared to be relatively rich in 1,8-cineole, limonene, *β*-pinene and *cis*-pinocamphone.

### 2.2. Methanol Extract Compositions and Contents of Total Polyphenols

The results of the liquid chromatography with diode array and mass spectrometry (LC-DAD-MS) analysis of methanol extracts (1E–6E) obtained from the tested samples of *H*. *officinalis* subsp. *aristatus* (1–6, Table 1) are presented in Table 3 and Table 4. LC-DAD-MS analysis of methanol extracts of hyssop flowering aerial parts revealed the presence of phenolic compounds, specifically benzoic acid derivative (syringic acid), hydroxycinnamic acid derivatives (chlorogenic, feruloylquinic and rosmarinic acids, as well as caffeoyl pentoside) and flavonoids (derivatives of quercetin and diosmetin). The identified compounds, their spectral characteristics and their retention times are given in Table 3. The comparative view of chromatograms of 1E–6E recorded at 320 nm is given in Supplementary Material Figure S1. It showed that chlorogenic and rosmarinic acids can be considered quantitatively dominant compounds based on their relative peak areas (%).

Identified phenolics were present in all extracts, regardless of the site of the plant material collection. Variability was reflected through relatively small differences in the concentrations of individual constituents. The content of chlorogenic acid was in the range between 23.35 and 33.46 mg/g, whereas rosmarinic acid was present in lower amounts (3.53–17.98 mg/g) (Table 4). Among the analyzed preparations, sample 4E was the richest in chlorogenic and rosmarinic acids.

The results are in good agreement with the literature data. Previous studies of ethanol and deodorized aqueous extracts of the aerial parts of wild-growing *H*. *officinalis* subsp. *aristatus* (originating from central Italy and eastern Serbia) showed the presence of chlorogenic acid, rosmarinic acid, 4-*O*-feruloylquinic acid and syringic acid [1,5]. Flavonoids, isoquercitrin (quercetin 3-*O*-glucoside) and diosmin (diosmetin 7-*O*-rutinoside), were also previously detected in extracts of hyssop herb [10,24]. In a study conducted by Borrelli et al. (2019), ethanol macerate of the hyssop aerial parts was chemically analyzed and the occurrence of caffeoyl pentoside, a hydroxycinnamate derivative, was confirmed [25]. In addition, a phenylethanoid glycoside martynoside was reported as a constituent of *H*. *seravshanicus* [26]. The findings of other authors regarding the quantitative composition of different extracts of hyssop herb are consistent with the presented results. Namely, Venditti et al. (2015), as well as Hatipoğlu et al. (2013), demonstrated that the content of chlorogenic acid is the highest among the quantities of phenolics [5,27]. Detailed analysis indicated that the contents of chlorogenic acid in the examined samples were 4–5 times higher than the corresponding values formerly reported, whereas the rosmarinic acid contents were closer to the literature values. However, certain variations can be expected and explained by a number of factors, e.g., differences in the extraction solvent used, the extraction methodology, the origin of the plant material and/or the developmental stage of the plant during collection.

The contents of total polyphenols (TPC) in tested samples ranged between 64.1 and 112.0 mg GAE/g (Table 4). The highest TPC was determined in sample 4E (112 mg GAE/g), whereas the lowest one was obtained in sample 3E (64.1 mg GAE/g). The sample richest in chlorogenic and rosmarinic acids was also the richest in total polyphenols. The order of the remaining extracts, by the decreasing TPCs, was: 5E > 1E > 6E > 2E.

Previous studies have yielded variable results, which is expected given that TPC can be affected by numerous factors. Namely, reported values for TPC in several different preparations of *H*. *officinalis* aerial parts were in a wide range, between 2.69 and 497.6 mg GAE/g [1,10,21,25,28].

### 2.3. Antioxidant Activity

Dry methanol extracts (1E–6E) of hyssop herb exhibited notable antioxidant activity in DPPH and FRAP assays (Table 5).

The lowest IC_50_ value, i.e., the best ability to neutralize DPPH radicals, was shown for the extract 4E (56.04 µg/mL), followed by 5E (79.37 µg/mL), whereas the lowest activity was observed in the case of 3E (199.89 µg/mL).

These results correlate well with the values of total antioxidant activity estimated by the FRAP assay. Namely, the highest FRAP value was obtained for the 4E extract (0.959 mmol Fe^2+^/g), followed by the 5E extract (0.877 mmol Fe^2+^/g), whereas the lowest value was demonstrated for the 3E extract (0.667 mmol Fe^2+^/g).

The data obtained in antioxidant assays correlate well with the contents of total polyphenols, which are known as constituents that contribute to the antioxidant activity of the plants. With regard to extracts 1E, 2E and 6E, there was no such strong link between the antioxidant activity and total polyphenol contents as there was with the aforementioned extracts. Compared to standard substances (rutin and ascorbic acid), tested hyssop preparations were less effective in DPPH radical scavenging and in the reduction of ferrous ion-2,4,6-tri(2-pyridyl)-*s*-triazine complex (Table 5).

Taking into account the presented results, it can be concluded that moderate antioxidant efficacy (IC_50_ < 100 μg/mL) was demonstrated for four of the six analyzed samples, with the best activity shown for sample 4E.

Literature reports on hyssop aerial parts preparations indicate considerable variability in IC_50_ values (25–2970 µg/mL) obtained in the DPPH test [1,10,25,28], which could be expected as the geographical origin of plant material, extraction procedures and antioxidant activity test protocols differ. With regard to the total antioxidant activity, Stanković et al. (2016) examined methanol extract of vegetative parts of *H. officinalis* from southeastern Serbia and found its FRAP value to be 0.73 mmol Fe^2+^/g [29]. The current study FRAP value is in good agreement with this reported value, as it ranged from 0.667 mmol Fe^2+^/g to 0.959 mmol Fe^2+^/g. The chemical composition may help explain the documented antioxidant activity. Namely, earlier published papers provide evidence that the dominant compounds of the tested extracts (chlorogenic and rosmarinic acids) exhibit significant efficacy in neutralizing DPPH radicals and reducing the ferrous ion complex [30,31,32,33,34].

### 2.4. Genotoxic and Antigenotoxic Activity

Potential genotoxicity and antigenotoxicity of methanol extracts and essential oils of hyssop herb were assessed using the Comet assay.

#### 2.4.1. Genotoxic Activity

Hyssop herb extracts did not exhibit a genotoxic effect at concentrations 100, 200 and 400 µg/mL (data not shown). With regard to the essential oils, the genotoxic effect did not manifest at the lowest tested concentration (2.5 µg/mL). These results were used to select concentrations for antigenotoxic activity testing.

#### 2.4.2. Antigenotoxic Activity

**Figure 3:**
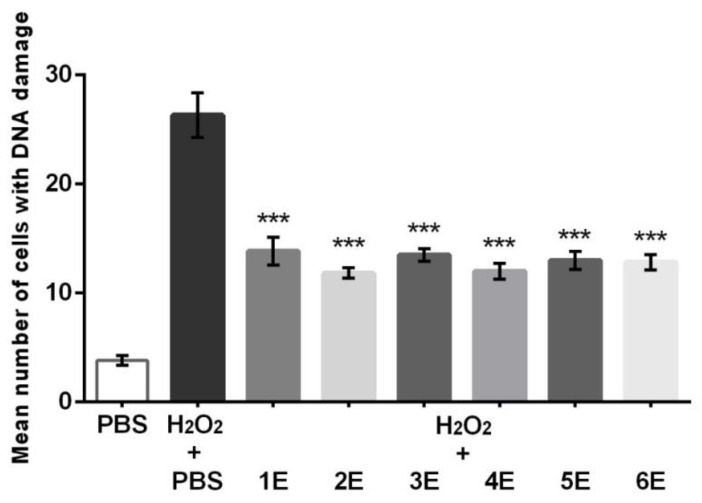
Antigenotoxic properties of methanol extracts of *H. officinalis* subsp. *aristatus* (1E–6E) against DNA damage in human peripheral blood leukocytes, induced by hydrogen peroxide (H_2_O_2_) in post-treatment protocol. Bars represent the mean value of cells with DNA damage ± standard error of the mean (SEM) versus the control treated with H_2_O_2_ (*n* = 3). PBS: phosphate-buffered saline solution. *** *p* < 0.0001.

At 400 µg/mL, all tested *H*. *officinalis* extracts significantly (*p* < 0.0001) reduced DNA damage in human peripheral blood leukocytes, induced by the pretreatment with hydrogen peroxide (Figure 3).

A decrease in the mean number of cells with DNA damage was the most pronounced for extracts 2E and 4E; however, there were no major differences in the antigenotoxic activity among the tested extracts. Similarly, Borrelli et al. (2019) indicated that the ethanol extracts of aerial parts of wild-growing *H. officinalis* subsp. *aristatus*, native to southern Italy, did not display genotoxicity, but counteracted DNA damage in Caco-2 cells caused by hydrogen peroxide [25]. The notable antigenotoxic activity of the tested extracts can be attributed at least in part to the significant content of polyphenols and their ability to neutralize free radicals. Chlorogenic and rosmarinic acids, as dominant compounds in the examined preparations, could be important for the observed activity, considering that earlier published data have shown that these compounds are effective in the Comet test [35,36].

**Figure 4:**
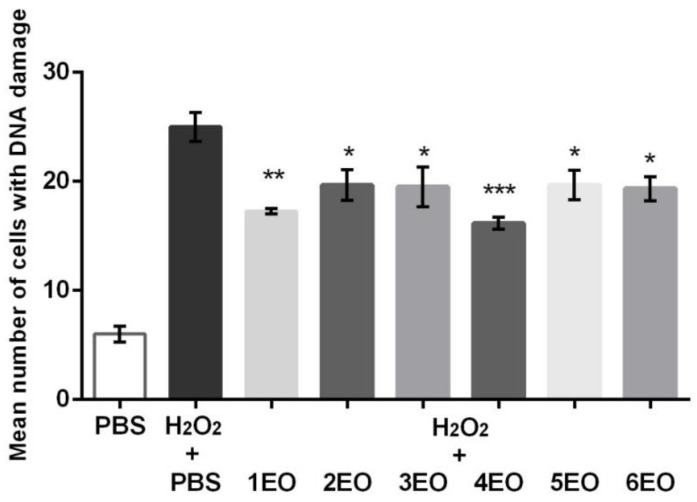
Antigenotoxic properties of essential oils of *H. officinalis* subsp. *aristatus* (1EO–6EO) against DNA damage in human peripheral blood leukocytes, induced by H_2_O_2_ in post-treatment protocol. Bars represent the mean value of cells with DNA damage ± standard error of the mean (SEM) versus control treated with H_2_O_2_ (*n* = 3). PBS: phosphate-buffered saline solution. * *p* < 0.01, ** *p* < 0.001, *** *p* < 0.0001.

The statistically significant antigenotoxic activity of the essential oils of hyssop aerial parts, applied at the concentration of 2.5 µg/mL, was revealed in the post-treatment. The best activity was shown for the essential oil 4EO (*p* < 0.0001), followed by commercial sample 1EO (*p* < 0.001), whereas other samples (2EO, 3EO, 5EO and 6EO) exhibited weaker, but statistically significant activity (*p* < 0.01) (Figure 4).

The dominant compound in the 4EO was phenylpropanoid methyl eugenol. With regard to the 1EO, 2EO, 5EO and 6EO samples, the dominant constituent was an oxygenated monoterpene 1,8-cineole, whereas *cis*-pinocamphone was present in the highest amount among components of 3EO. For methyl eugenol [37] and 1,8-cineole [38], there are literature data that confirm their ability to neutralize free radicals, which could contribute to the antigenotoxic effect. The beneficial action of essential oils could be based on their participation in the direct neutralization of free radicals, but also their contribution to DNA molecule repair.

These results show that hyssop extracts and essential oils exhibit statistically significant antigenotoxic activity. Therefore, conduction of in vivo tests is needed to estimate the potential of *H*. *officinalis* preparations more reliably.

### 2.5. Cytotoxic Activity

A cytotoxic agent can induce cell death when it leads to cell demise or, on the other hand, it can cause reproductive cell death, inhibiting cell growth and proliferation, while the cell remains alive. This study aimed to determine the overall cytotoxic potential, cytostatic and cytocidal effects of extracts (1E–6E) against human tumor cell lines (SW480, MDA-MB 231 and HeLa). Additionally, these effects were examined on non-transformed human lung fibroblast cell line (MRC-5).

Cytotoxic effects of these extracts were examined in a range of seven concentrations after 24, 48 and 72 h of treatment by 3-(4,5-dimethylthiazol-2-yl)-2,5 diphenyltetrazolium bromide dye (MTT) colorimetric assay. MTT assay is a method that indirectly determines cell viability. MTT is a water-soluble yellow-colored crystal that easily passes through the cell membrane because of its positive charge. In metabolically active cells, MTT is reduced to non-soluble purple formazan crystals. Mitochondrial reductase (succinate dehydrogenase), active only in viable cells, catalyzes this reaction, so the reduction of the original compound to formazan is directly proportional to the number of viable cells. The obtained results are represented as dose-response curves (Supplementary Figure S2) and IC_50_, SI (Table 6) parameters.

The data revealed that extracts 1E–6E displayed a statistically significant percentage of growth inhibition in a dose-dependent manner on all designated cell lines after 48 h and 72 h (*p* < 0.05); however, that trend was not noticed after 24 h of treatment (*p* > 0.05). Time-dependent growth inhibition was present only on the HeLa cell line with high statistical significance (*p* < 0.0001), while a significant time-dependent effect was revealed only at the highest examined concentration on MRC-5 and MDA-MB 231 cells (*p* < 0.05). On the other hand, the increase in growth inhibition of the SW480 cell line was independent of the exposure period.

To evaluate the overall inhibitory potential of the examined extracts, we calculated IC_50_ as a parameter of growth inhibition in relation to the control, which did not take into account the initial cell number at time zero. Examined extracts showed very low overall inhibitory activity against healthy cell line MRC-5, but also against the SW480 and MDA-MB 231 tumor cell lines, because their IC_50_ values exceeded the highest examined concentration (data not shown). On the other hand, the HeLa cell line was susceptible to their effect with high overall inhibition indicated by low IC_50_ values. Extracts 2E and 4E exhibited the strongest overall inhibitory activity after 48 h and 72 h of treatment, followed by extracts 1E, 5E and 6E, while extract 3E had the highest IC_50_. Regardless, there was no statically significant difference among the tested extracts. Importantly, the extracts displayed activity highly selective for HeLa cells with selectivity index (SI) values that ranged between 8 and 20. Antitumor activity of most clinically applied agents is restricted because of their large spectrum of side effects and general toxicity, including to some normal cells. Although scientists continue to develop compounds with a targeted mechanism of action, many of those compounds still lack selectivity for tumor cells [39]. In that term, natural products are considered as less toxic for normal cells and as a biologically friendly approach, as evidenced by the large number of extracts and secondary metabolites in clinical trials [40].

Further, according to National Cancer Institute (NCI) recommendations [41], we calculated three parameters to disclose whether the examined extracts had cytostatic (GI_50_, TGI) or cytocidal (LC_50_) effects on designated cell lines (Table 6). The calculated parameters showed low to absent cytostatic or cytocidal activity of tested extracts 1E–6E against the SW480 and MDA-MB 231 cancer cell lines and, importantly, against the non-transformed cell line MRC-5 (data not shown). Contrarily, on the HeLa cell line, all examined extracts acted as very potent inhibitors of net cell growth with very low GI_50_ values, especially extracts 2E and 4E, which exhibited a net cell growth inhibition for 50% of cells at concentrations lower than the minimum concentration examined after 72 h of treatment (GI_50_ < 0.3 μg/mL). Extracts 1E, 5E and 6E exhibited a net cell growth inhibition of 50%, with similar potency as the previous extracts. Compared to the extract of commercial hyssop herb (1E), only extract 3E had lower cell growth inhibition activity with a high statistical significance (*p* < 0.0001). The same trend was present in the perspective of total growth inhibition and cytocidal activity. Namely, extracts 2E and 4E provoked strong cytostatic effect after 72 h of treatment with TGI values of 1.69 μg/mL and <0.3 μg/mL, respectively. Also, LC_50_ values of these extracts were significantly lower than for other extracts (*p* < 0.05), indicating their potent cytocidal nature. Extracts 1E, 5E and 6E followed the same trend. The tumor grows when the total rate of division of its cells exceeds the total mortality rate. The ability to grow uncontrollably is gained through the accumulation of mutations of genes that manage cell proliferation and cell death. Therefore, the agents that can override these defects, stop uncontrolled cell division and kill cancer cells are beneficial in cancer treatment. Tested extracts 1E, 2E and 4E–6E showed, along with high selectivity, a potent ability both to inhibit cell proliferation and to induce cell death in a human cervical cancer cell line. Therefore, those extracts and their compounds should be further examined for their possible application in the therapy of this type of cancer.

The dominant compounds in the extracts are, as mentioned above, chlorogenic and rosmarinic acids, whose cytotoxic potential has been reported earlier [42,43]. The extract 4E had the highest content of chlorogenic and rosmarinic acids, as well as total phenolic compounds, while the extract 3E, which exhibited the weakest cytotoxic activity compared to other tested extracts, had the lowest contents of total phenols and rosmarinic acid. On the other hand, the extract 2E, which also gave very good results in this study, together with the extract 4E, was distinguished neither by the content of total phenols nor by chlorogenic or rosmarinic acids. The extracts 2E and 4E also showed antigenotoxic activity in the comet assay (post-treatment protocol). Therefore, we can conclude that chlorogenic and rosmarinic acid probably contributed to the overall cytotoxic potential of the methanol extract of hyssop herb. The contribution of individual components of the extract and/or their synergistic/additive action to selective cytotoxicity against HeLa cells is of particular interest and should be further investigated in the future.

## 3. Material and Methods

### 3.1. Chemicals and Reagents

The acetonitrile and methanol for the chemical analysis and antioxidant activity testing were from J.T. Baker Chemicals Co. (Phillipsburg, NJ, USA); formic acid, 2,4,6-tris(2-pyridyl)-s-triazine (TPTZ), 2,2-diphenyl-1-picrylhydrazyl (DPPH), ferrous sulphate, Folin–Ciocalteu (FC) reagent, ferric chloride and gallic acid were purchased from Sigma-Aldrich (St. Louis, MO, USA); L-ascorbic acid was procured from Acros (Geel, Belgium), whereas rosmarinic acid (≥99%), chlorogenic acid (>97%) and rutin were supplied by Carl Roth (Karlsruhe, Germany). Solvents used for the LC-DAD-MS analysis were of LC-MS grade, whereas the other solvents and reagents were of analytical purity.

For determination of genotoxic and antigenotoxic activity, phosphate-buffered saline solution (PBS) was purchased from Fisher Scientific (Pittsburgh, PA, USA); hydrogen peroxide was purchased from Zorka Pharma (Šabac, Serbia), low-melting-point agarose (LMPA) and normal-melting-point agarose (NMPA) were purchased from Sigma-Aldrich (St. Louis, MO, USA).

For the investigation of cytotoxic activity, Dulbecco’s modified Eagle medium (DMEM), heat-inactivated fetal bovine serum (FBS), L-glutamine, non-essential amino acids, dimethyl sulfoxide (DMSO), penicillin and streptomycin, as well as a trypsin and ethylenediaminetetraacetic acid (EDTA) combination dissolved in phosphate-buffered saline (PBS) were purchased from Sigma Aldrich (St. Louis, MO, USA). Finally, 3-(4,5-dimethylthiazol-2-yl)-2,5 diphenyltetrazolium bromide dye (MTT) was purchased from Sigma Aldrich (St. Louis, MO, USA).

### 3.2. Plant Material

The flowering aerial parts of *Hyssopus officinalis* subsp. *aristatus* (Godr.) Nyman were collected from five localities in the territory of Montenegro (Table 1).

Identification of the plant material was carried out according to the *Flora Europaea* [44]. Voucher specimens (Table 1) were deposited in the herbarium of the Faculty of Natural Sciences and Mathematics in Podgorica (Montenegro), Department of Biology (TGU). The collected plant material was air-dried at room temperature.

In addition, a commercial sample of hyssop herb was purchased from a local production enterprise in Serbia. Commercial material was produced from the wild-growing plants collected from the sites located in southeastern Serbia (Pirot and Nišava Districts).

Prior to hydrodistillation or extraction, dried plant material was ground to a coarse powder.

### 3.3. Essential Oil Isolation

Essential oils were isolated from the plant material by hydrodistillation in a clevenger-type apparatus, according to Procedure I of the *Pharmacopoea Jugoslavica IV* (1984), suitable for isolation of essential oils lighter than water [45].

For calculating the yield of essential oils (%, based on the dry weight of the plant material), five consecutive volumetric determinations were performed.

The essential oils were kept at 4 °C in amber glass vials that were tightly closed and protected from light.

### 3.4. Extraction Procedure

Methanol extracts were prepared by bimaceration, according to the *Pharmacopoea Jugoslavica IV* (1984) [45]. Obtained methanol extracts were brought to dryness using a rotary evaporator under reduced pressure and a temperature below 50 °C, and subsequently, by a stream of nitrogen. Prior to analysis, the extracts were stored at 4 °C in tightly closed glass jars. The extracts were reconstituted just before the analysis by the addition of methanol up to the concentration of 5 mg/mL and filtered through a 0.45 μm membrane filter (Captive Syringe Filters, Agilent, Germany).

### 3.5. GC-MS Analysis of Essential Oils

Qualitative and semi-quantitative chemical analysis of essential oils was performed by gas chromatography coupled with mass spectrometry (GC-MS) on an Agilent Technologies 6890 Series gas chromatograph.

A 0.2 µL aliquot of each essential oil solution (10 µL/mL in hexane) was injected in split mode, with a split ratio of 1:20, at the temperature 220 °C. The components were separated on a nonpolar poly (tetramethyl-1,4-sulfenylenesiloxane) HP-5ms column (Agilent Technologies; 30 m × 0.25 mm, layer thickness 0.25 µm). The column was eluted in the temperature-programmed mode: initial temperature 60 °C, increase 3 °C/min to 246 °C (total analysis time 62 min). High-purity helium (5.0) was used as the carrier gas with a constant flow of 0.9 mL/min. The effluent was transferred to the Agilent Technologies 5975 series electron ionization mass spectrometer via a transfer line maintained at 280 °C. The parameters of the mass spectrometer were as follows: electron energy 70 eV, ion source temperature 230 °C, quadrupole temperature 150 °C. Acquisition scan mode was applied in the range *m/z* 35–400 with a solvent delay of 2.30 min. To achieve a better agreement between the experimental and library spectra, the standard spectra tune was used.

Acquired data were processed using Agilent Technologies MSD ChemStation software (revision E01.01.335) in combination with NIST MS Search software (ver. 2.0d). The spectral libraries Wiley Registry of Mass Spectral Data [46], NIST/EPA/NIH Mass Spectral Library [47] and Adams’ mass spectral library of essential oils (3rd Edition) [48] were used to identify mass spectra. The identity of the compounds was demonstrated by comparing the MS spectra and linear retention indices with the literature data. The relative proportion of compounds was determined using the area standardization method and expressed as area %.

### 3.6. LC-DAD-MS Analysis of Methanol Extracts

Liquid chromatography with diode array and mass spectrometry (LC-DAD-MS) was carried out on Agilent LC/MS System 1260/6130 (Agilent Technologies, Waldbronn, Germany), equipped with ChemStation software Rev. B.04.03-SP1, a degasser (model G1311B), a quaternary pump (G1311B/1260), an autosampler (G1329B), a diode array detector (DAD) (G4212B), a single quadrupole atmospheric pressure ionization - electrospray ionization (API-ESI) mass selective detector (MSD) (6130) and a reverse-phase column Zorbax SB-Aq (150 × 3.0 mm; particle diameter 3.5 µm, Agilent Technologies), maintained at an operating temperature of 25 °C.

The mobile phase consisted of 0.1% aqueous formic acid (phase A) and acetonitrile (phase B). The following gradient elution program was used: 10% B to 35% B (0–20 min), 35% B to 90% B (20–24 min), 90% B (24–25 min) and 90% B to 10% B (25–30 min), at a total operating time interval of 30 min, a mobile phase flow rate of 0.35 mL/min and an injection volume of 3.00 µL. UV spectral data of all peaks were collected in the range 190 to 640 nm, and chromatograms were recorded at 210, 270, 320 and 350 nm. API-ESI in the negative polarity and the range *m/z* 100–1000 was used for analyte ionization. The parameters of the ion source were as follows: fragmentation voltages 100 and 250 V, drying gas flow (nitrogen) 10.0 L/min, drying gas temperature 350 °C, nebulization pressure 40 psi and capillary voltage 3500 V.

The compounds were identified by comparing their UV and MS spectral data, and the retention times (Rt) with the corresponding data obtained for the standard compounds under the same chromatographic conditions, as well as by comparing the data with previously published literature data.

The contents of chlorogenic and rosmarinic acids were determined using the external standard method at 320 nm.

Stock solutions of chlorogenic acid (0.4 mg/mL) and rosmarinic acid (0.5 mg/mL) were prepared by dissolving the reference substances in methanol and their subsequent filtration through a syringe filter (0.45 µM, Captiva, Agilent). By further dilution with the same solvent, calibration standards with a wide range of concentrations were prepared. This procedure (including the preparation of stock solutions) was repeated three times so that three measurements were made for each calibration point. Calibration curves and coefficients of determination were obtained by linear regression analysis. Limit of detection (LoD) and limit of quantification (LoQ) were calculated according to the International Conference on Harmonization guidelines [49].

Chlorogenic acid calibration curve (y = 26733x + 70.594, R^2^ = 0.9998; linearity range 0.02–0.4 mg/mL; LoD = 0.005 mg/mL; LoQ = 0.015 mg/mL) and rosmarinic acid (y = 18000x + 39.94, R^2^ = 0.9998; 0.00625–0.5 mg/mL; LoD = 0.003 mg/mL; LoQ = 0.010 mg/mL) were used for contents determination.

### 3.7. Total Phenols

The contents of total phenolic compounds in dry methanol extracts were determined using Folin–Ciocalteu (FC) reagent according to the method described by Velioglu et al. [50]. The results were expressed as mg of gallic acid equivalents (GAE) per g of dry extract (mg GAE/g) and represent the mean value of the three consecutive measurements.

### 3.8. Antioxidant Activity

The antioxidant activity of investigated extracts was assessed by a set of two commonly used tests: DPPH radical scavenging assay and FRAP assay.

#### 3.8.1. DPPH Assay

The DPPH radical scavenging assay was carried out according to the methodology described by Kukić et al. [51] with slight adaptations that were necessary for conducting the assay on microtiter plates.

The methanol solutions of the tested hyssop extracts were prepared in different concentrations, along with the standard methanol solutions of rutin. The test solution consisted of a mixture of 0.1 mL of the methanol solution of tested extract, 0.1 mL of methanol and 0.05 mL of 0.5 mM methanol solution of DPPH. The mixtures were shaken vigorously and incubated for 30 min in the dark at room temperature. The absorbances were measured at 492 nm against methanol as a blank test on Biochrom EZ Read 400 microtiter plate reader. The negative control consisted of 0.2 mL of methanol and 0.05 mL of 0.5 mmol/L DPPH solution. DPPH inhibition was calculated according to the following formula:

I (%) = (Ac − At)/Ac × 100(1)

where Ac is the absorbance of the control and At is the absorbance of test solutions.

The results are expressed as half-maximum inhibitory concentration (IC_50_ values; µg/mL) values, which denote the concentrations that neutralize 50% of DPPH radicals and are the mean values of the three consecutive determinations.

#### 3.8.2. FRAP Assay

The total antioxidant activity of dry methanol extracts of hyssop herb and standard solutions was determined using the ferric reducing/antioxidant power (FRAP) assay, essentially as described by Pellegrini et al. (2003) [52].

In brief, the test, standard (rutin, 0.1 mg/mL; ascorbic acid, 0.05 mg/mL) or control solutions were transferred (0.1 mL) into test tubes and 3.0 mL of ex tempore prepared FRAP reagent (25 mL acetate buffer, 300 mmol/L, pH 3.6 + 2.5 mL 10 mmol/L TPTZ in 40 mmol/L HCl + 2.5 mL 20 mmol/L FeCl_3_ × 6H_2_O) was added. The absorbances were recorded at 593 nm against a blank containing 0.1 mL of solvent after 30 min incubation at 37 °C. FRAP values were calculated from the calibration curve of FeSO_4_ × 7H_2_O solutions, covering the concentration range between 100 and 1000 μmol/L, and expressed as mmol Fe^2+^/g dry extract (mmol Fe^2+^/g). All measurements were performed in triplicate.

### 3.9. Genotoxic and Antigenotoxic Activity

Peripheral blood samples from three volunteer subjects (21–35 years of age), were collected using a method of finger extraction into heparinized containers and immediately subjected to the experiment. The subjects were non-smokers and they had taken neither medications nor alcohol and dietary supplements. They signed their written consent, in accordance with the regulations of the ethical standards of the Ethics Committee for Biomedical Investigations at the Faculty of Pharmacy in Belgrade.

Dry methanol extracts of the hyssop herb were dissolved in the phosphate-buffered saline solution (PBS); the essential oils were dissolved in absolute ethanol and then diluted with PBS.

#### 3.9.1. Genotoxic Activity Assay

Human whole blood cells (WBC) were incubated with different concentrations of essential oils (12.5, 5 and 2.5 µg/mL) or methanol extracts of hyssop herb (100, 200 and 400 µg/mL) for 30 min at 37 °C. Tested concentrations were selected on the basis of the literature data [53,54]. The samples were treated in parallel with a negative control of PBS for 30 min at 37 °C, as well as with a positive control of hydrogen peroxide (H_2_O_2_, 50 µM) for 20 min at 4 °C and PBS for 30 min at 37 °C. A concentration of 50 μM H_2_O_2_ was the lowest one that induced a statistically significant increase of DNA damage in the tested cells.

#### 3.9.2. Antigenotoxic Activity Assay

The antigenotoxic activity of extracts or essential oils (which did not show activity in the genotoxicity assay) was analyzed in the post-treatment [55,56]. WBC were treated with H_2_O_2_ for 20 min at 4 °C and washed with PBS; afterward, they were incubated for 30 min at 37 °C with tested essential oils/methanol extracts. The concentration of 400 µg/mL was chosen for further testing, because it was the most effective concentration in the antigenotoxic assessment of commercial extract. The attenuation of H_2_O_2_-induced DNA damage in human peripheral blood leukocytes in the post-treatment with EO was assessed using the concentration that did not induce a statistically significant increase of DNA damage in the tested cells in the genotoxicity assessment (2.5 µg/mL).

#### 3.9.3. Comet Assay

The comet assay (single-cell gel electrophoresis), which is normally used to detect DNA damage, was performed according to the protocol described by Singh et al. (1988), called the alkaline method, according to which DNA breaks and alkaline labile sites are detected, while the degree of DNA damage is indicated by the extent of DNA molecule migration [57].

A quantity of 100 μL of the test suspension, containing 6 μL of peripheral blood suspended in 0.67% of LMPA, was evenly applied to the prepared microscopic plates coated with a layer of 1% NMPA. Cover glasses were placed on the plates and then left at 4 °C for 5 min. The cover glasses were slowly removed and the cells were exposed to the appropriate treatment (as previously described for genotoxic or antigenotoxic activities). After treatment, the third layer of 100 μL of 0.5% LMPA was applied, cover glasses were placed and the plates were again left at 4 °C for 5 min. The cover glasses were again carefully removed, the microscope plates were immersed in lysis solution (2.5 M NaCl, 100 mM EDTA, 10 mM Tris, 1% Triton X100 and 10% DMSO; pH 10 adjusted with NaOH) and kept at 4 °C overnight. After cell lysis, DNA denaturation was performed in the same solution that was later used for electrophoresis for 30 min (10 M NaOH, 200 mM EDTA, pH ≥ 13).

Electrophoresis was performed at 25 V and 300 mA for 30 min. After electrophoresis, alkali neutralization in gels was performed by rinsing twice with a neutralizing buffer (0.4 M Tris, pH 7.5) for 10 min, and then with distilled water. After rinsing, the comet was stained and visualized with fluorescent dye-ethidium bromide solution (20 μg/L). After staining (15 min), the plates were ready for analysis.

All experiments were done three times, in duplicate.

To determine the degree of DNA damage for each donor and each concentration, 200 nucleoids (comets) were selected and analyzed by random selection, or 100 from each duplicate microscope plate, using an Olympus BX 50 fluorescent microscope (Olympus Optical Co., GmbH, Hamburg, Germany) with a mercury short arc lamp (HBO) (50 W, 516–560 nm Carl Zeiss Microscopy, Jena, Germany) at 100 × magnification.

The analysis was performed by determining the length and density of DNA in the “tail” based on which of the nucleoids (comets) were classified into five groups, as described by Anderson et al. (1994) [58]: A—without the “tail” (<5% DNA damage), B—low degree of damage (5–20%), C—medium degree of damage (20–40%), D—high degree of damage (40–95%) and E—complete DNA damage (>95%). The degree of total DNA damage was expressed as the sum of all DNA damage/migrations over 5% (B + C + D + E).

### 3.10. Investigation of Cytotoxic Activity of Hyssop Herb

#### 3.10.1. Cell Lines and Cultures

The potential cytotoxic effect of methanolic extracts was examined on the human cervix (HeLa), breast (MDA-MB 231) and colon (SW480) cancer cell lines, while their selectivity was tested on non-transformed human lung fibroblasts (MRC-5). Cell lines were obtained from American Type Culture Collection (ATCC).

Cells were cultured in DMEM at pH = 7.4, enriched with 10% heat-inactivated FBS, L-glutamine, non-essential amino acids (0.1 mM), penicillin (100 IU/mL) and streptomycin (100 μg/mL). Cultivation was performed in T-25 flasks (ThermoFisher Scientific, Waltham, MA, USA) in an aseptic environment under standard culture conditions (37 °C, absolute humidified air and 5% CO_2_). The media were changed when necessary and cells were subcultured every fifth day. Just before in vitro experiments, subconfluent cell monolayers (~80%) in the logarithmic growth phase were detached from the bottom of the flask by short-term treatment with 0.25% trypsin and 0.53 mM EDTA combination dissolved in phosphate-buffered saline.

#### 3.10.2. Extract Solutions

The stock solutions were prepared by dissolving methanol extracts in DMSO at 50 mg/mL and stored at 4 °C. Preceding the treatment, fresh working solutions of the extracts at different concentrations were prepared by diluting stock solution in supplemented DMEM. The final DMSO concentration in working solutions was lower than 0.5% (*v/v*).

#### 3.10.3. MTT Assay

The cytotoxic potential of the extracts against HeLa, MDA-MB 231, SW480 and MRC-5 was evaluated in vitro by MTT assay as a common colorimetric technique for cell viability determination [59].

The cells were seeded in 96-well flat-bottom microtiter plates (ThermoFisher Scientific, Waltham, MA, USA) at a density of 5 × 10^3^ cells per well and incubated overnight to adhere. After 24 h, the supernatant was replaced with extract solutions at seven different concentrations (0.3, 1, 3, 10, 30, 100 and 300 μg/mL). In the control wells, cells grew in the presence of supplemented nutrient media only. The cells were incubated for 24, 48 and 72 h. The MTT solution, at a final concentration 0.5 mg/mL in the unsupplemented medium, was added to each well at time zero (after overnight incubation) and the end of different incubation periods. Following 2 h of incubation, the MTT solution was discarded and formazan crystals were solubilized with 150 μL of DMSO. The plates were shaken for 5 min and the absorbance was measured at 550 nm with a multiplate reader (Zenyth 3100, Anthos Labtec Instruments GmbH, Wals-Salzburg, Austria).

All experiments were repeated at least three times in triplicate.

#### 3.10.4. Cytotoxicity Parameters

The results of the MTT assay are presented as the percentage of the values for control cells that was arbitrarily set to 100%. Cell growth inhibition was calculated according to the expression:

(A_0_ − A) × 100/A_0_ (2)

where A_0_ is absorbance from control wells and A is absorbance from wells exposed to the tested extracts.

The measure of the overall inhibitory activity of the agent was evaluated through IC_50_, which is defined as the concentration of the agent that inhibits the biological activity of the target cells by 50%.

The selectivity index (SI) was calculated as the quotient of IC_50_ values for the treated non-transformed cell line and the IC_50_ values for the tested extracts on malignant cells. SI < 2 indicates general toxicity of a compound, SI ≥ 2 indicates selective toxicity and SI ≥ 3 indicates highly selective toxicity [60].

Following the NCI recommendations [41], GI_50_, TGI and LC_50_ parameters were calculated for each extract:

- The GI_50_ value is the concentration where 100 × (T − T0)/(C − T0) equals 50 and measures the growth inhibitory power of the examined extracts;
- The TGI value is the concentration of the tested extract where 100 × (T − T_0_)/(C − T_0_) equals 0 and measures the cytostatic effect;
- The LC_50_ value is the concentration of the drug where 100 × (T − T_0_)/T_0_ equals 50 and measures the cytotoxic effect of extracts.

In these formulas, T_0_ is the absorbance at time zero (when the compound is added), Tis the absorbance of the test well after 24, 48 or 72 h of exposure to the test compound and C is the optical density of the control wells (cells incubated for 48 h with no additives). If the effect was not reached or was exceeded, the value for that parameter was expressed as greater or less than the maximum or minimum concentration tested.

### 3.11. Statistics

The results of the experiments are represented as mean ± standard deviation.

Principal component analysis (PCA) and hierarchical cluster analysis (HCA) using Statistica^®^ v.8.0 (www.statsoft.com, accessed on 1 April 2021) and StatistiXL^®^ Version 2.0 add-in for MS Excel^®^ (www.statistixl.com, accessed on 1 April 2021) were applied to examine the interrelationships between the chemical compositions of the essential oils.

The results of LC-MS quantitative analysis and assays on antioxidant activity were analyzed by SPSS software (version 20.0) using one-way ANOVA and post hoc Tukey’s test. Differences between the mean values were considered statistically significant if *p* < 0.05.

The IC_50_, GI_50_, TGI and LC_50_ parameters were calculated using MS Office Excel^®^ free add-in ED50 plus v1.0 software (www.sciencegateway.org/protocols/cellbio/drug/data/, accessed on 1 April 2021). SPSS software version 20 was used for statistical data analysis. The Shapiro–Wilk test was used to test the normality of data distribution. Depending on the results normality test, for the comparison of groups, one-way analysis of variance (ANOVA) or its non-parametric equivalent Kruskal–Wallis test was used.

For the genotoxic and antigenotoxic activity assays, the results are expressed as the mean value (*n* = 3) ± standard error of the mean (SEM). Statistical analysis of the comet assay results was performed using one-way analysis of variance (ANOVA) with Tukey’s post hoc test for comparisons of different treatments vs. the respective controls. GraphPad Prism 6.0 software was used. A regression was used to determine the effect of the bioactive substance concentration on the outcome. The values of the obtained data were considered statistically significant if *p* < 0.05, and statistically highly significant if *p* < 0.001.

## 4. Conclusions

This study evaluated the antioxidant activity and genotoxicity/antigenotoxicity, as well as cytotoxic, cytostatic and cytocidal effects against human tumor and non-transformed human lung fibroblast cell lines of the investigated *Hyssopus officinalis* essential oils and/or extracts, with respect to their chemical composition. Our results revealed high variability in the composition of essential oils, as three chromatographic profiles of the investigated essential oils of wild-growing plants from Montenegro could be distinguished: oils rich in 1,8-cineole and relatively rich in *β*-pinene, but low in *cis*-pinocamphone; oils rich in *β*-pinene, limonene, *cis*-pinocamphone and methyl eugenol, but relatively low in 1,8-cineol; and oils relatively rich in 1,8-cineole, limonene, *β*-pinene and *cis*-pinocamphone. The essential oil from the commercial plant material from Serbia, being rich in 1,8-cineole and *β*-pinene, but low in *cis*-pinocamphone, appeared similar to only one of the samples obtained from wild-growing plants from Montenegro. Both the extracts and the essential oils significantly reduced in vitro DNA damage. In addition, potent and selective cytotoxic action of the hyssop methanol extracts on the HeLa cell line was observed. These findings deserve closer attention and our further investigations, which will be performed in the prospective future, and should be directed to the panel of cancer cell lines derived from the most sensitive tissue (cervix), along with a detailed mechanism of antitumor effect and the isolation/chemical characterization of the constituents that are presumably responsible for observed activity.

## Figures and Tables

**Table 1:** Plant material: origin, collection data and yields of essential oils and extracts. ^a^ Values are the means of five consecutive determinations.

Sample	Plant Material Origin	Site of Collection	Geographic Coordinates	Altitude (m)	Habitat	Collection Date (dd/mm/yyyy)	Voucher Specimen	Essential Oil (EO) Yield (mL/100 g) ^a^	MeOH Extract (E) Yield (% *w/w*)
1	Commercial sample (Serbia)	Southeastern Serbia	N/A	N/A	N/A	N/A	N/A	1.00	12.02
2	Wild-growing (Montenegro)	Kuči	N 42°31′55′′ E 19°24′07′′	870	rocky	13/09/2018	1420263	0.40	9.48
3	Wild-growing (Montenegro)	Šavnik	N 42°57′16′′ E 19°05′59′′	880	rocky pasture	19/09/2018	1420261	0.54	10.24
4	Wild-growing (Montenegro)	Piva	N 43°9′25′′ E 18°50′46′′	750	rocky	14/09/2018	1420162	0.65	9.05
5	Wild-growing (Montenegro)	Piperi	N 42°34′23′′ E 19°16′0.8′′	800	rocky pasture	07/09/2018	1420259	0.79	10.21
6	Wild-growing (Montenegro)	Cuce	N 42°35′19′′ E 18°47′40′′	820	rocky	12/09/2018	1420260	0.48	9.64

**Table 2:** Essential oil composition of *Hyssopus officinalis* subsp. *aristatus*. * Arithmetic retention index.

Amount (%)								
t_R_ [min]	AI *	Compound	1EO	2EO	3EO	4EO	5EO	6EO
5.517	925	*α*-Thujene	0.00	0.51	0.00	1.05	1.44	0.00
5.706	932	*α*-Pinene	2.08	4.13	1.12	0.53	0.79	1.03
6.762	972	Sabinene	1.86	1.24	0.57	0.47	0.54	0.56
6.872	976	*β*-Pinene	6.73	9.13	16.33	15.79	9.69	5.48
7.238	990	*β*-Myrcene	0.93	0.46	0.46	0.00	0.43	0.36
8.343	1024	*p*-Cymene	0.27	1.92	0.00	0.00	0.00	0.28
8.482	1028	Limonene	7.99	7.99	16.11	23.81	21.77	15.43
8.569	1030	1,8-Cineole	67.10	42.07	9.77	1.42	38.19	56.08
8.765	1036	*Z*-*β*-Ocimene	3.57	2.94	2.06	1.88	3.11	3.06
9.142	1046	*E*-*β*-Ocimene	0.27	0.00	0.00	0.00	0.00	0.00
9.531	1057	*γ*-Terpinene	0.31	0.58	0.00	0.00	0.00	0.00
12.592	1138	*trans*-Pinocarveol	0.23	2.26	0.83	0.54	0.00	0.61
13.463	1159	*trans*-Pinocamphone	0.00	1.84	3.34	8.34	4.72	0.00
13.556	1162	Pinocarvone	0.00	1.20	3.99	1.67	0.00	0.41
14.027	1173	*cis*-Pinocamphone	1.15	5.61	22.75	14.72	14.54	0.00
14.961	1196	Myrtenal	0.32	3.71	1.02	0.66	0.69	0.80
20.403	1325	Myrtenyl acetate	0.00	1.25	0.00	0.00	0.00	0.00
22.856	1384	*β*-Bourbonene	0.00	0.00	0.00	0.00	0.00	0.31
23.758	1406	Methyl eugenol	5.43	0.00	19.24	28.33	3.52	13.70
24.265	1418	*E*-*β*-Caryophyllene	0.47	0.00	0.00	0.00	0.00	0.00
26.771	1480	Germacrene D	0.40	0.00	0.00	0.00	0.00	0.36
Monoterpene hydrocarbons			24.01	28.9	36.65	43.53	37.77	26.2
Oxygenated monoterpenes			68.8	57.94	41.7	27.35	58.14	57.9
Sesquiterpene hydrocarbons			0.87	0.00	0.00	0.00	0.00	0.67
Phenylpropanoids			5.43	0.00	19.24	28.33	3.52	13.70
Total identified			99.11	86.84	97.59	99.21	99.43	98.47

**Table 3:** Assignment, retention times, UV and MS spectral data of phenolic compounds in methanol extracts of *Hyssopus officinalis* subsp. *aristatus*. ^a^ Identification by comparing with commercial reference compounds. ^b^ Tentative identification by comparing acquired UV and MS spectral data with literature data.

Peak No.	t_r_ (min)	UV λ_max_ (nm)	ESI-MS Data (*m/z*)	Assignment
1	4.255	280	395.1 [2M-H]^−^, 197 [M-H]^−^, 153.1	Syringic acid ^b^
2	9.107	218, 240, 298 sh, 326	707.1 [2M-H]^−^, 353.1 [M-H]^−^, 191	Chlorogenic acid (5-*O*-caffeoylquinic acid) ^a^
3	10.954	218, 238, 298 sh, 328	623.1 [2M-H]^−^, 311.1 [M-H]^−^, 134.1	Caffeoyl pentoside ^b^
4	12.087	218, 238, 296 sh, 326	735.2 [2M-H]^−^, 367.1 [M-H]^−^, 173.1	Feruloylquinic acid ^b^
5	15.339	256, 266 sh, 356	463.1 [M-H]^−^, 300.1	Quercetin *O*-hexoside ^b^
6	17.215	252, 266, 348	607.2 [M-H]^−^, 299.1, 284	Diosmetin *O*-deoxyhexosyl-hexoside ^b^
7	18.461	286 sh, 328	719.1 [2M-H]^−^, 359 [M-H]^−^, 197, 161.1	Rosmarinic acid ^a^

**Table 4:** Content of total phenols, chlorogenic acid and rosmarinic acid in methanolic extracts of *Hyssopus officinalis* subsp. *aristatus*. Different letters in the superscript indicate statistically different values at *p* < 0.05.

Sample	Total Phenols (mg GAE/g)	Chlorogenic Acid (mg/g)	Rosmarinic Acid (mg/g)
1E	74.7 ± 0.8 ^c^	23.35 ± 0.2 ^a^	13.71 ± 0.19 ^d^
2E	68.2 ± 0.8 ^b^	30.94 ± 0.11 ^d^	5.35 ± 0.02 ^b^
3E	64.1 ± 1.3 ^a^	24.12 ± 0.11 ^b^	3.53 ± 0.03 ^a^
4E	112.0 ± 1.6 ^e^	33.46 ± 0.08 ^e^	17.98 ± 0.25 ^e^
5E	81.8 ± 0.8 ^d^	33.17 ± 0.1 ^e^	4.97 ± 0.12 ^b^
6E	69.0 ± 0.3 ^b^	30.19 ± 0.1 ^c^	8.13 ± 0.04 ^c^

**Table 5:** Total antioxidant activity and 2,2-diphenyl-1-picrylhydrazyl (DPPH) radical scavenging activity in methanolic extracts of *Hyssopus officinalis* subsp. *aristatus*. Different letters in the superscript indicate statistically different values at *p* < 0.05.

Sample	DPPH-IC_50_ (μg/mL)	FRAP mmol Fe^2+^/g
1E	88.42 ± 3.48 ^d^	0.815 ± 0.012 ^b^
2E	175.41 ± 2.92 ^e^	0.781 ± 0.012 ^a,b^
3E	199.89 ± 0.60 ^f^	0.667 ± 0.004 ^a^
4E	56.04 ± 0.19 ^b^	0.959 ± 0.003 ^c^
5E	79.37 ± 1.51 ^c^	0.877 ± 0.007 ^b,c^
6E	87.90 ± 0.67 ^d^	0.736 ± 0.023 ^a,b^
Rutin	4.67 ± 1.41 ^a^	4.111 ± 0.0253 ^d^
Ascorbic acid	-	8.181 ± 0.136 ^e^

**Table 6:** Concentrations of extracts (µg/mL) that induce inhibition of biological activity in 50% cells (IC_50_), 50% growth inhibition (GI_50_), total growth inhibition (TGI) and 50% lethality (LC_50_) in the HeLa cell line, expressed as X ± SD. SI: selectivity index.

HeLa		1E	2E	3E	4E	5E	6E
IC_50_	24 h	>100	>100	>100	>100	>100	>100
	48 h	22.72 ± 3.53	16.97 ± 2.10	44.38 ± 1.96	16.74 ± 1.43	25.90 ± 4.60	25.32 ± 7.80
	72 h	19.53 ± 1.03	15.15 ± 1.72	33.43 ± 1.36	14.97 ± 0.78	18.73 ± 0.53	20.04 ± 5.10
SI	24 h	0.97	1.52	1.71	1.31	1.10	3.61
	48 h	14.19	20.14	12.08	19.61	8.34	11.87
	72 h	12.17	13.87	8.30	15.04	11.31	7.82
GI_50_	24 h	6.95 ±0.95	6.00 ± 0.34	98.09 ± 11.08	5.56 ± 0.18	7.67 ± 1.45	7.46 ± 0.99
	48 h	4.91 ± 0.84	3.49 ± 0.46	65.64 ± 3.66	2.54 ± 0.45	5.61 ± 1.22	5.75 ± 0.68
	72 h	0.86 ± 0.51	<0.3	59.38 ± 1.85	<0.3	4.97 ± 0.54	4.76 ± 0.52
TGI	24 h	16.90 ± 4.96	14.67 ± 0.58	>100	12.91 ± 0.44	47.25 ± 5.66	27.55 ± 1.89
	48 h	13.60 ± 1.75	11.00 ± 0.56	>100	9.18 ± 0.59	19.90 ± 4.38	16.77 ± 1.52
	72 h	13.27 ± 1.33	1.69 ± 0.36	>100	<0.3	18.42 ± 3.57	13.63 ± 1.26
LC_50_	24 h	>100	>100	>100	>100	>100	>100
	48 h	61.09 ± 16.15	35.65 ± 1.16	>100	27.07 ± 1.56	63.66 ± 2.30	41.15 ± 6.75
	72 h	43.19 ± 10.03	26.02 ± 2.88	>100	20.28 ± 1.10	60.65 ± 1.59	31.20 ± 5.93

## Data Availability

Data available upon request.

## Supplementary Materials

The following are available online at https://www.mdpi.com/article/10.3390/plants10040711/s1, Figure S1: A comparative view of LC-DAD chromatograms of methanol extracts (1E–6E) of *H. officinalis* recorded at 320 nm, Figure S2: Dose-response curves in MTT assay after 24, 48 and 72 h treatment of MRC-5 (a–f), SW480 (g–l), MDA-MB 231 (m–r) and HeLa (s–x) cell lines with extracts 1E–6E, Table S1: Component loadings and score coefficients for constituents of *Hyssopus officinalis* essential oils.
