# Supplementary material for: Antioxidant, Antigenotoxic and Cytotoxic Activity of Essential Oils and Methanol Extracts of Hyssopus officinalis L. Subsp. aristatus (Godr.) Nyman (Lamiaceae)
Source: Plants (Basel). 2021 Apr 7;10(4):711. doi: 10.3390/plants10040711 (PMC8067569; doi:10.3390/plants10040711)
Supplement: Supplementary file 1 [file plants-10-00711-s001.pdf]

Supplementary materials

Antioxidant, Antigenotoxic and Cytotoxic Activity of Essential Oils and Methanol Extracts of *Hyssopus officinalis* L. subsp. *aristatus* (Godr.) Nyman (Lamiaceae)

Tijana Mićović[1], Dijana Topalović[2], Lada Živković[2], Biljana Spremo-Potparević[2], Vladimir Jakovljević[3,4], Sanja Matić[5], Suzana Popović[6], Dejan Baskić[6,7], Danijela Stešević[8], Stevan Samardžić[9], Danilo Stojanović[10] and Zoran Maksimović[9*]

[1] Institute for Medicines and Medical Devices of Montenegro, Bulevar Ivana Crnojevića 64a, Podgorica, Montenegro

[2] Department of Pathobiology, Faculty of Pharmacy, University of Belgrade, Vojvode Stepe 450, Belgrade, Serbia

[3] Department of Physiology, Faculty of Medical Sciences, University of Kragujevac, Svetozara Markovića 69, Kragujevac, Serbia

[4] Department of Human Pathology, First Moscow State Medical University „I.M.Sechenov", Moscow, Russian Federation

[5] Department of Pharmacy, Faculty of Medical Sciences, University of Kragujevac, Svetozara Markovića 69, Kragujevac, Serbia

[6] Department of Microbiology and Immunology, Center for Molecular Medicine and Stem Cell Research, Faculty of Medical Sciences, University of Kragujevac, Svetozara Markovića 69, Kragujevac, Serbia

[7] Public Health Institute, Nikole Pašića 1, Kragujevac, Serbia

[8] Faculty of Natural Sciences and Mathematics, University of Montenegro, Džordža Vašingtona bb, Podgorica, Montenegro;

[9] Department of Pharmacognosy, Faculty of Pharmacy, University of Belgrade, Vojvode Stepe 450, Belgrade, Serbia

[10] Department of Botany, Faculty of Pharmacy, University of Belgrade, Vojvode Stepe 450, Belgrade, Serbia

[*] Correspondence: zmaksim1@pharmacy.bg.ac.rs

Figure S1. A comparative view of LC-DAD chromatograms of methanol extracts (1E–6E) of *H. officinalis* recorded at 320 nm. CA – chlorogenic acid, RA – rosmarinic acid.

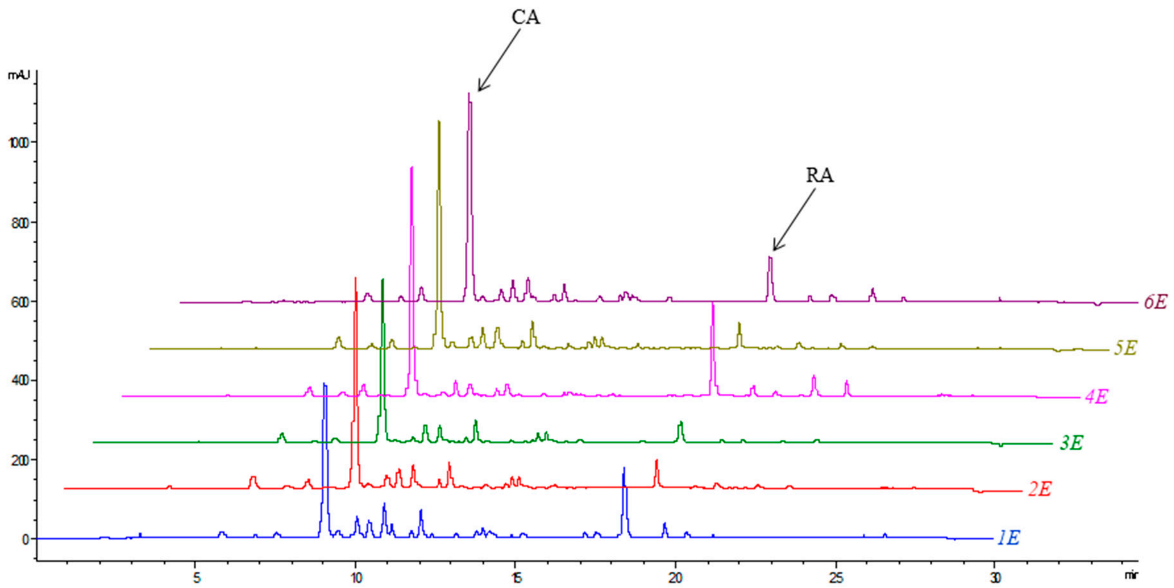

Figure S2. Dose-response curves in MTT assay after 24, 48 and 72 h treatment of MRC-5 (a - f), SW480 (g - l), MDA-MB 231 (m - r) and HeLa (s - x) cell lines with extracts 1E-6E. The values are presented as mean ± SD of triplicates from at least three independent experiments.

Figure S2(a)

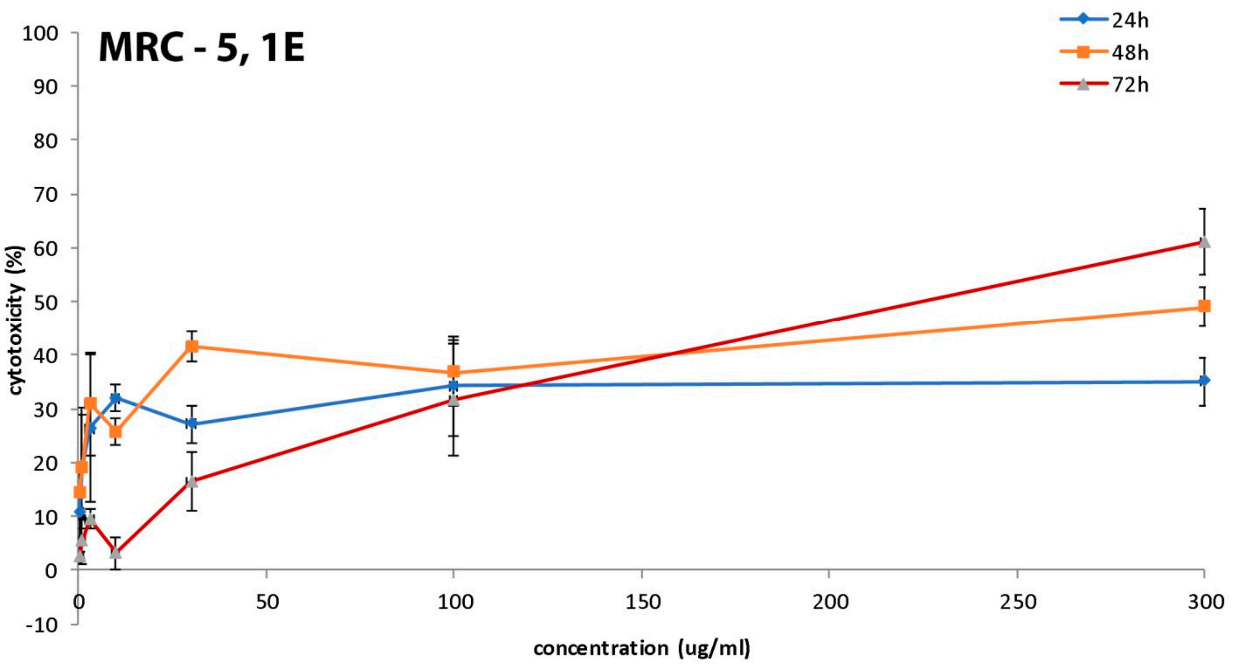

Figure S2(b)

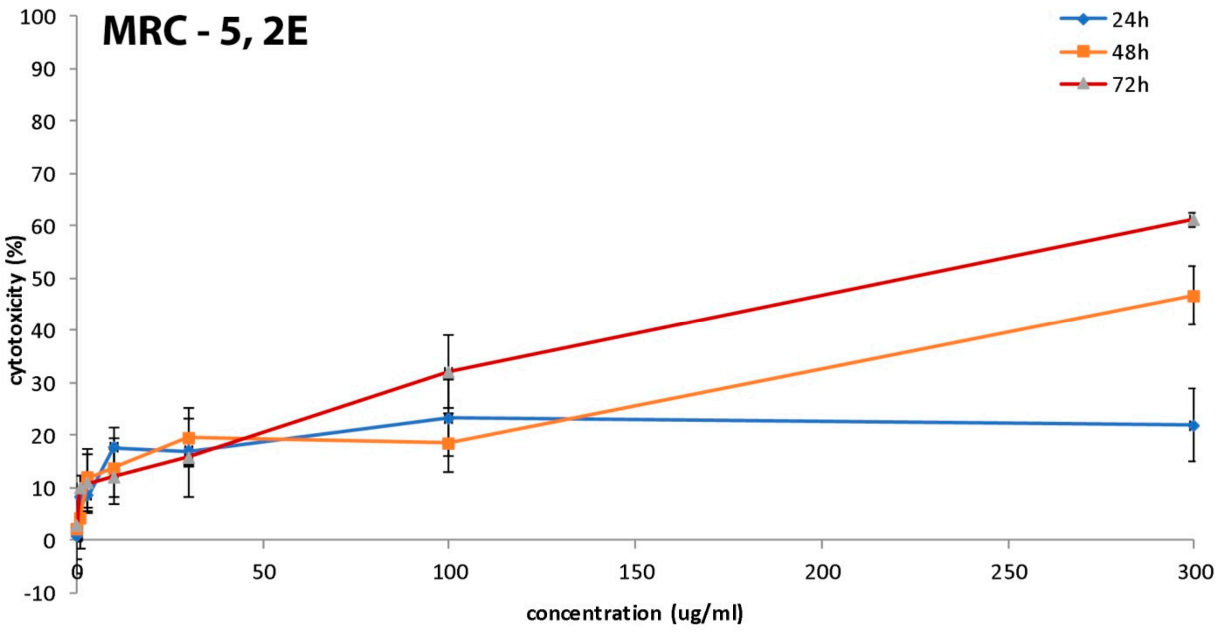

Figure S2(c)

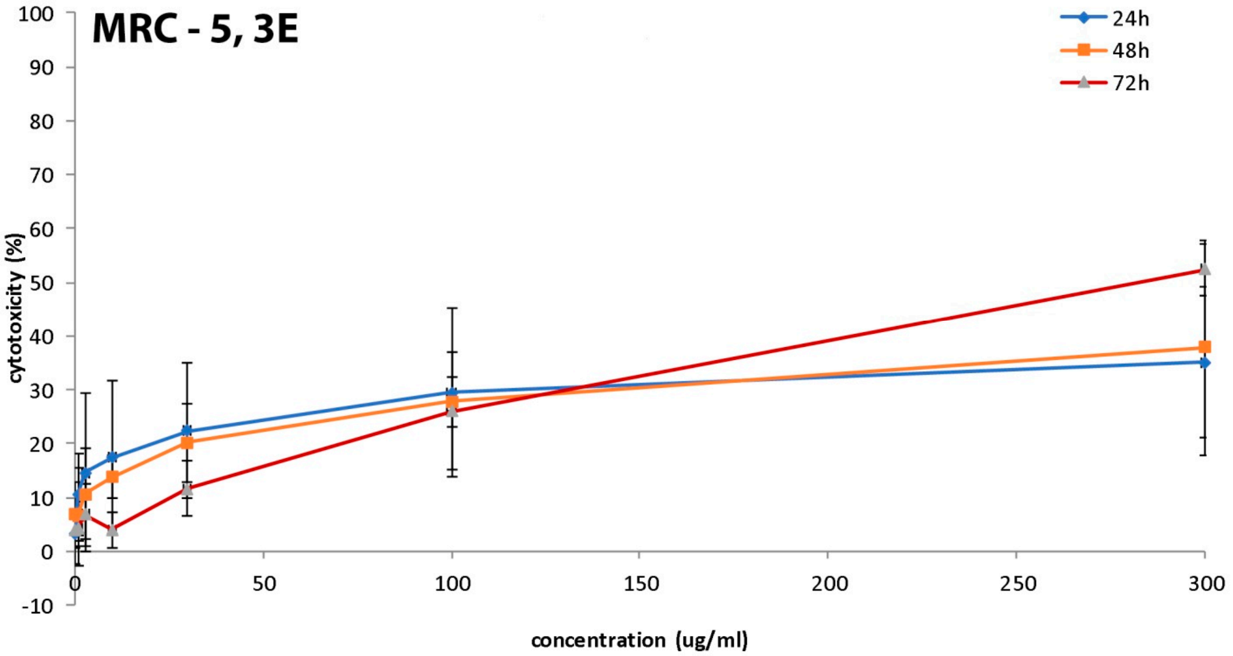

Figure S2(d)

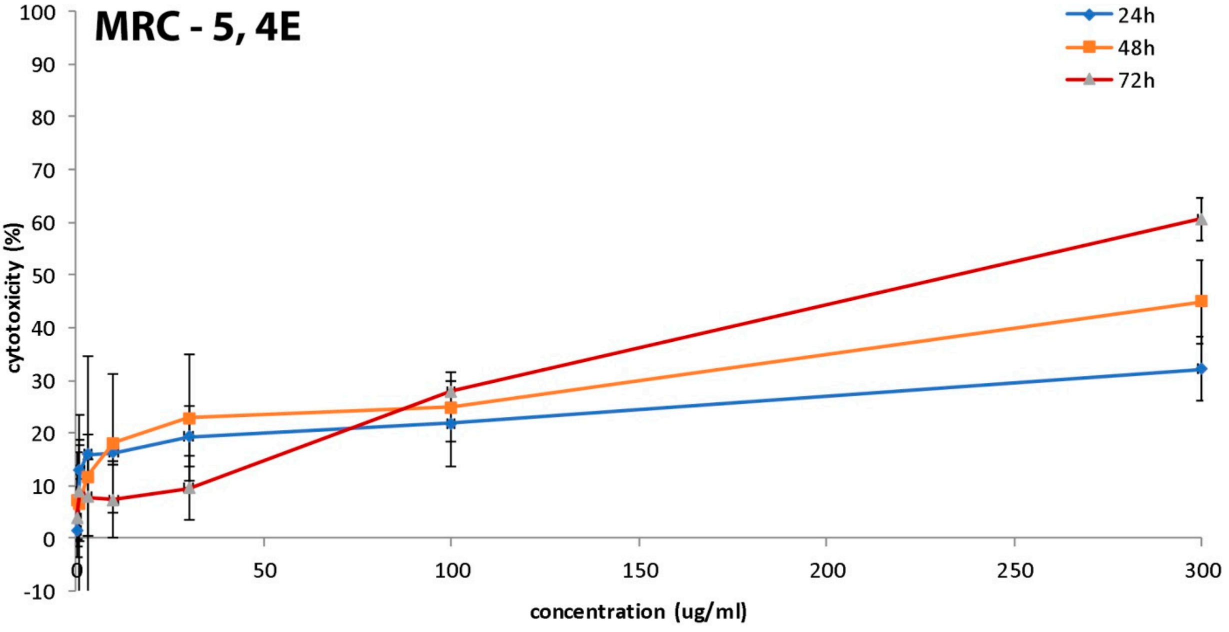

Figure S2(e)

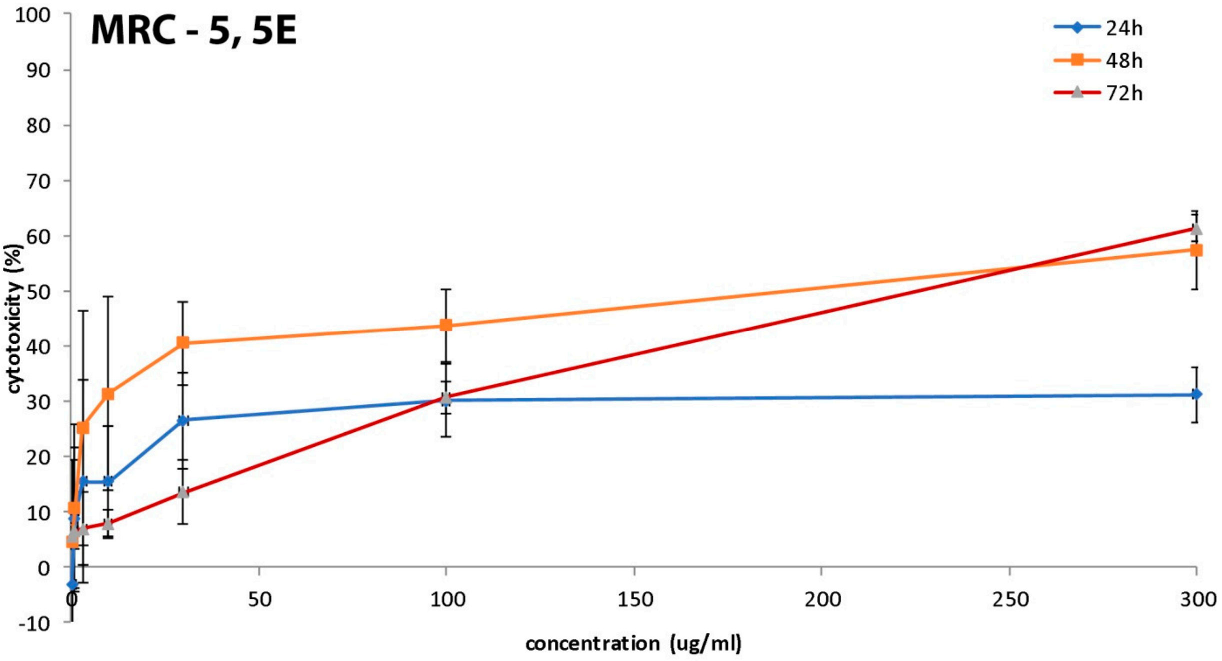

Figure S2(f)

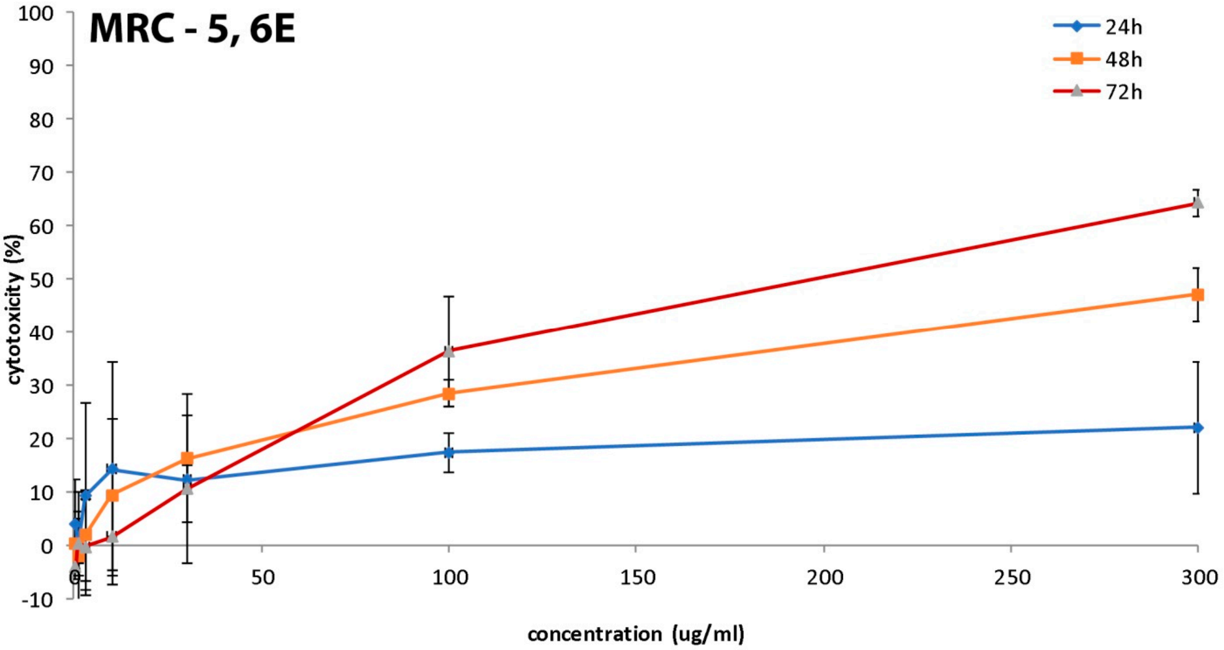

Figure S2(g)

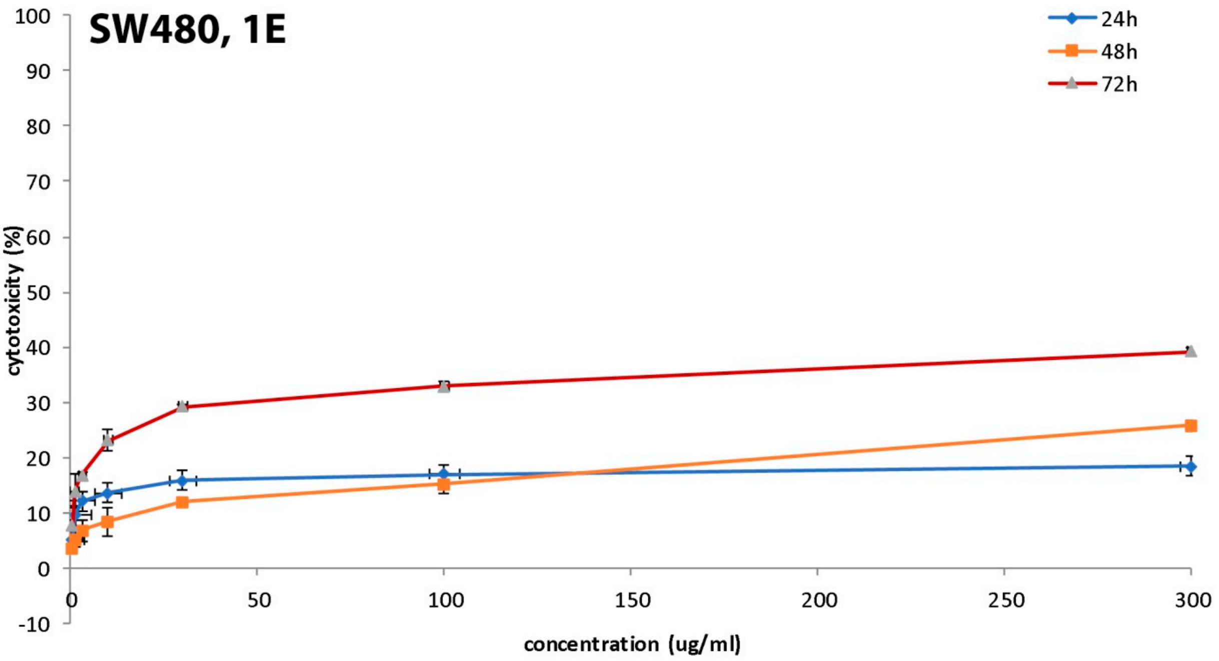

Figure S2(h)

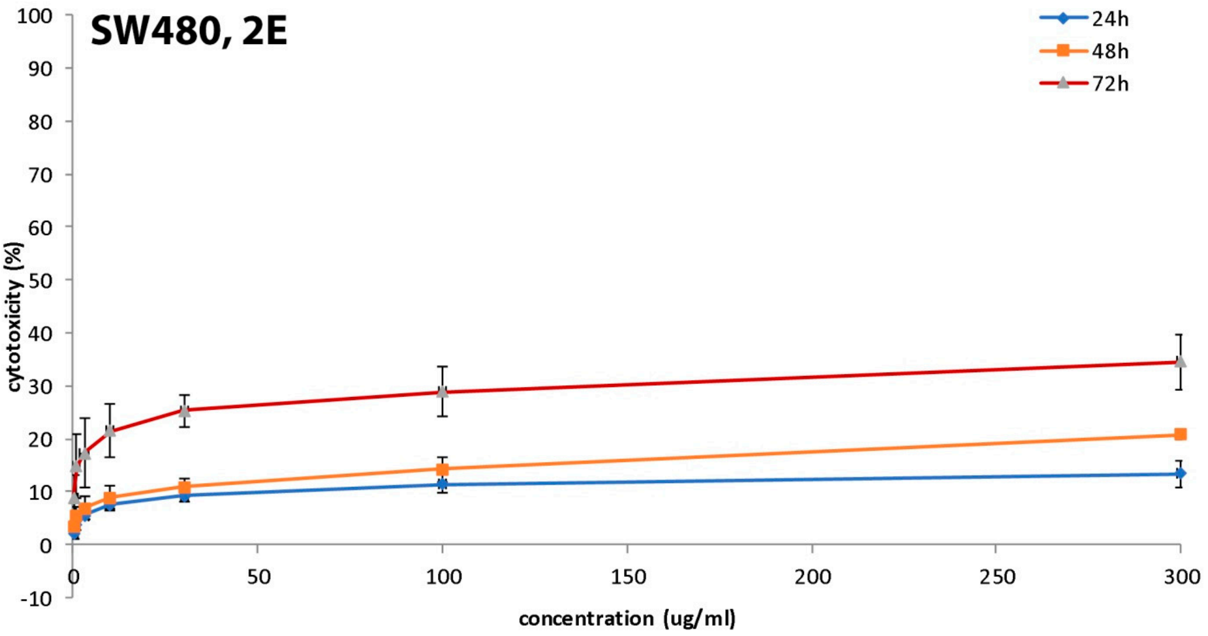

Figure S2(i)

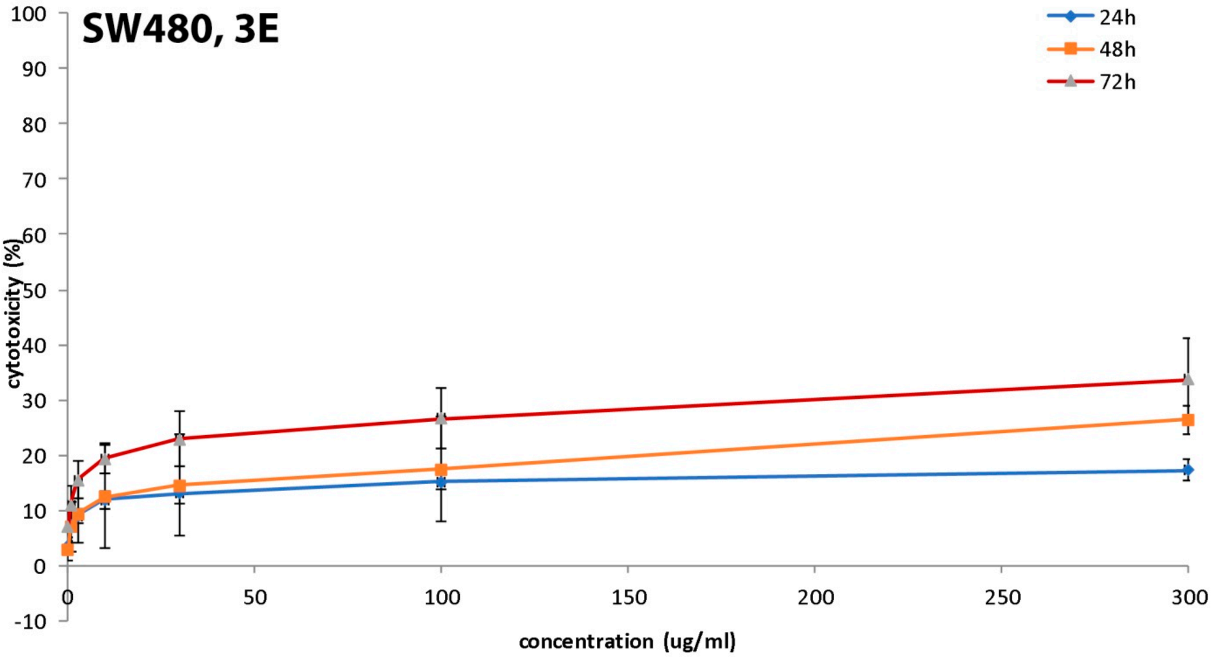

Figure S2(j)

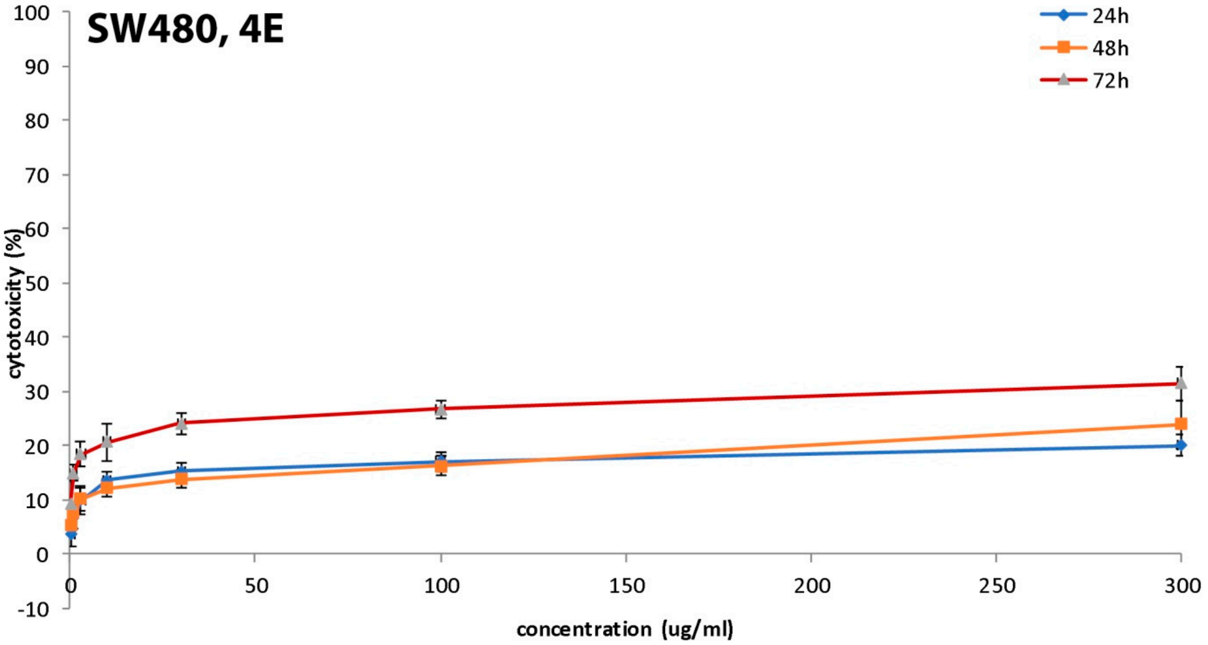

Figure S2(k)

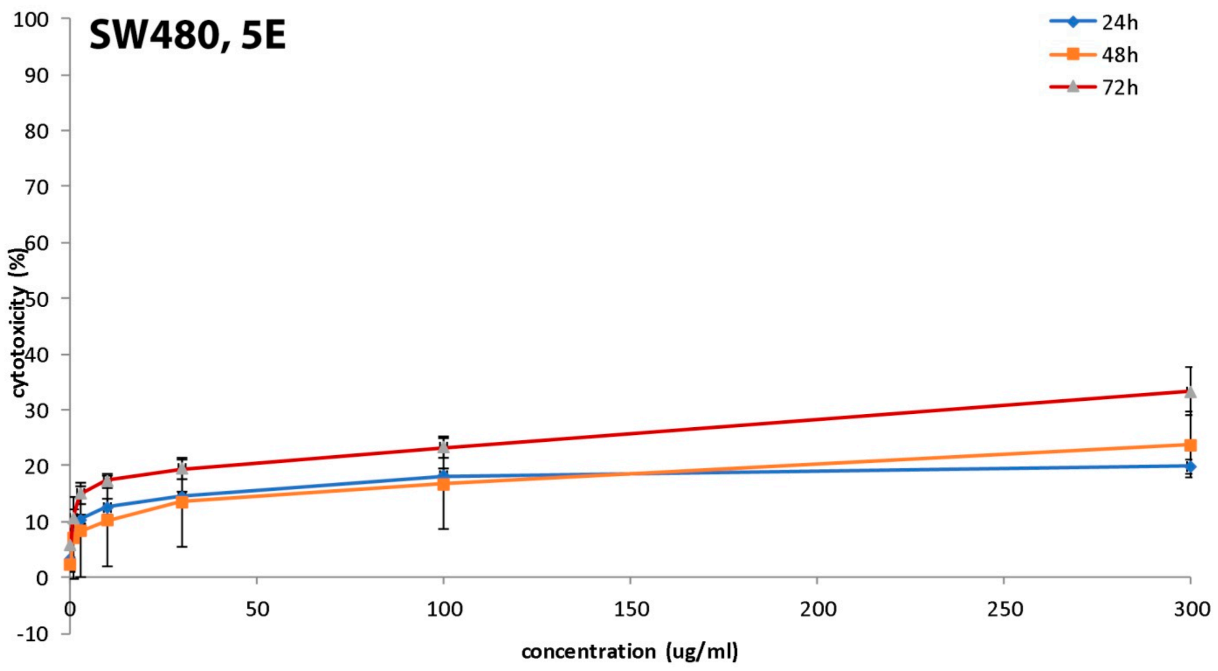

Figure S2(l)

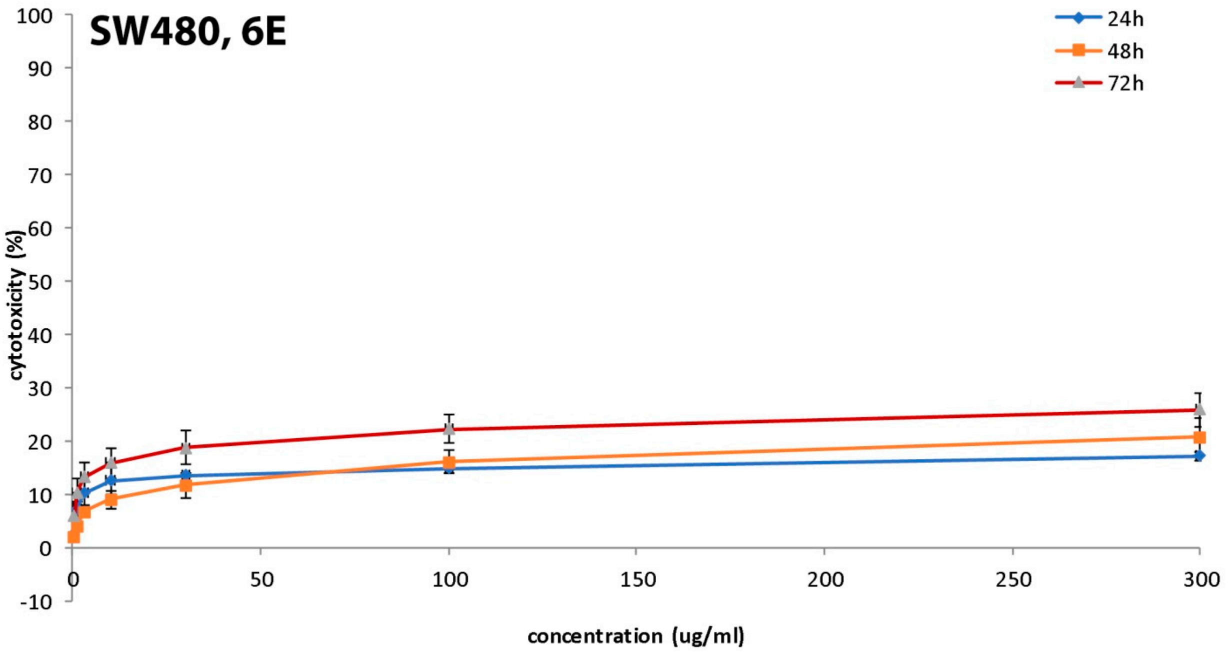

Figure S2(m)

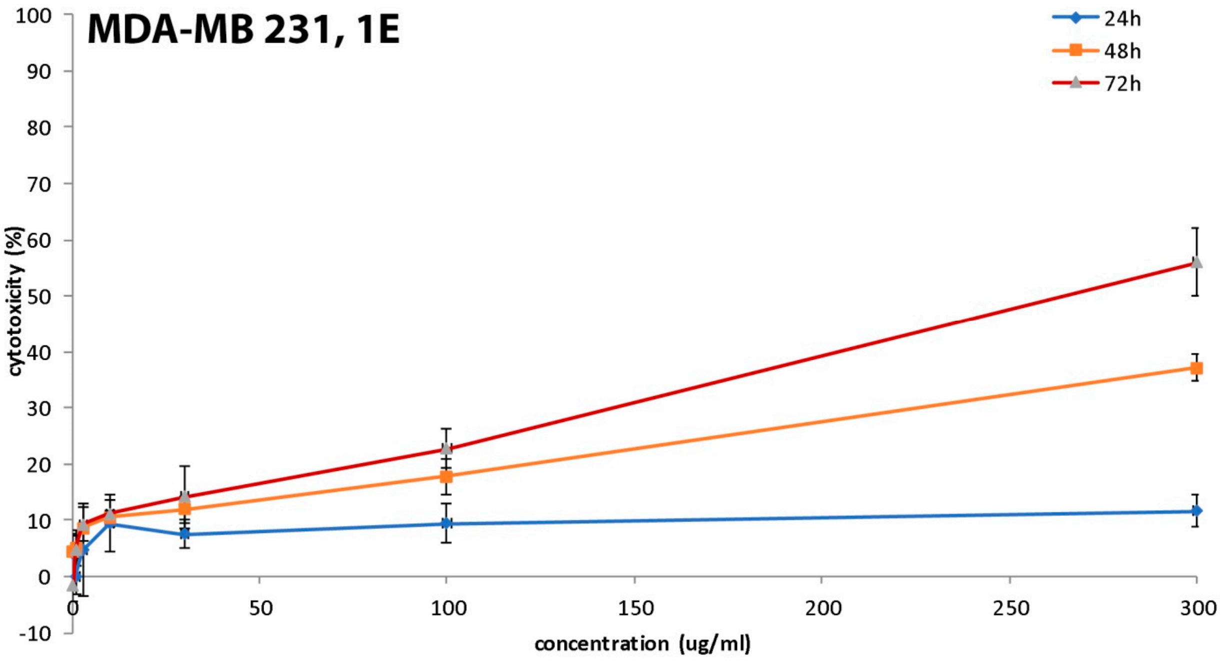

Figure S2(n)

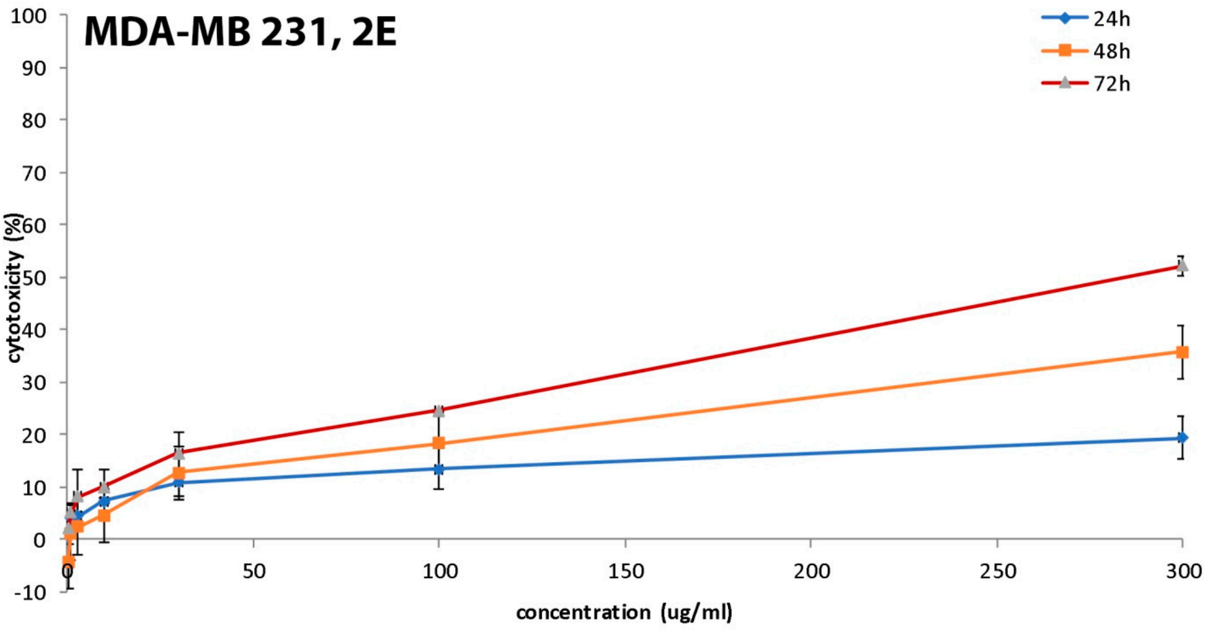

Figure S2(o)

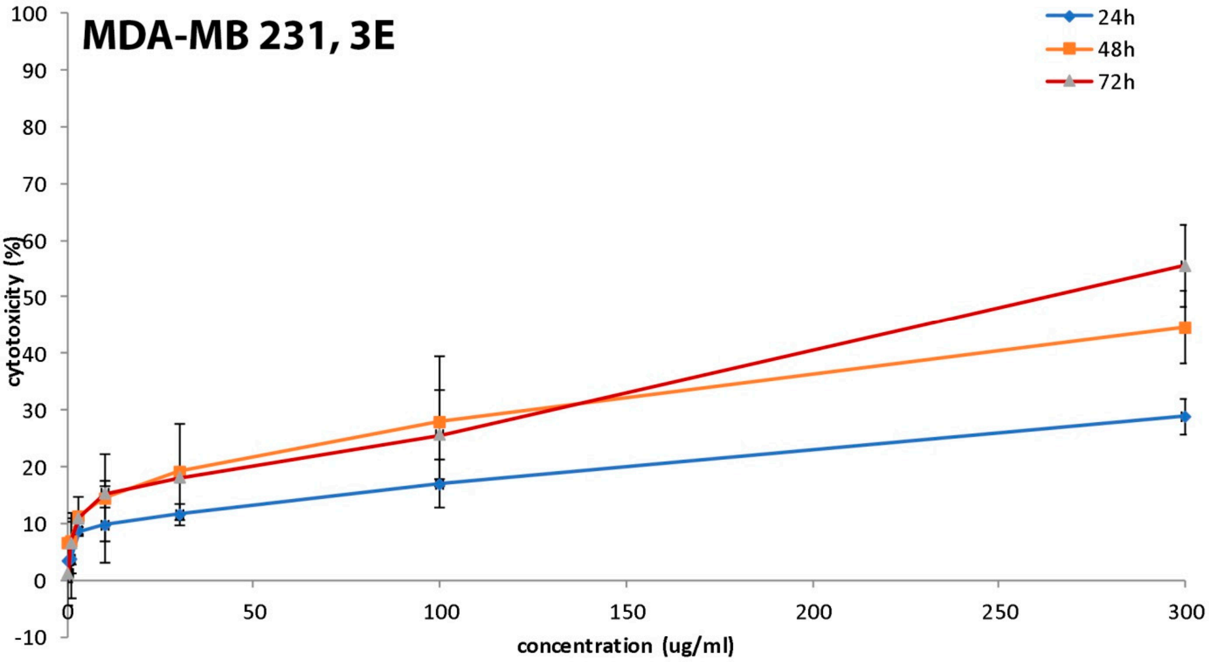

Figure S2(p)

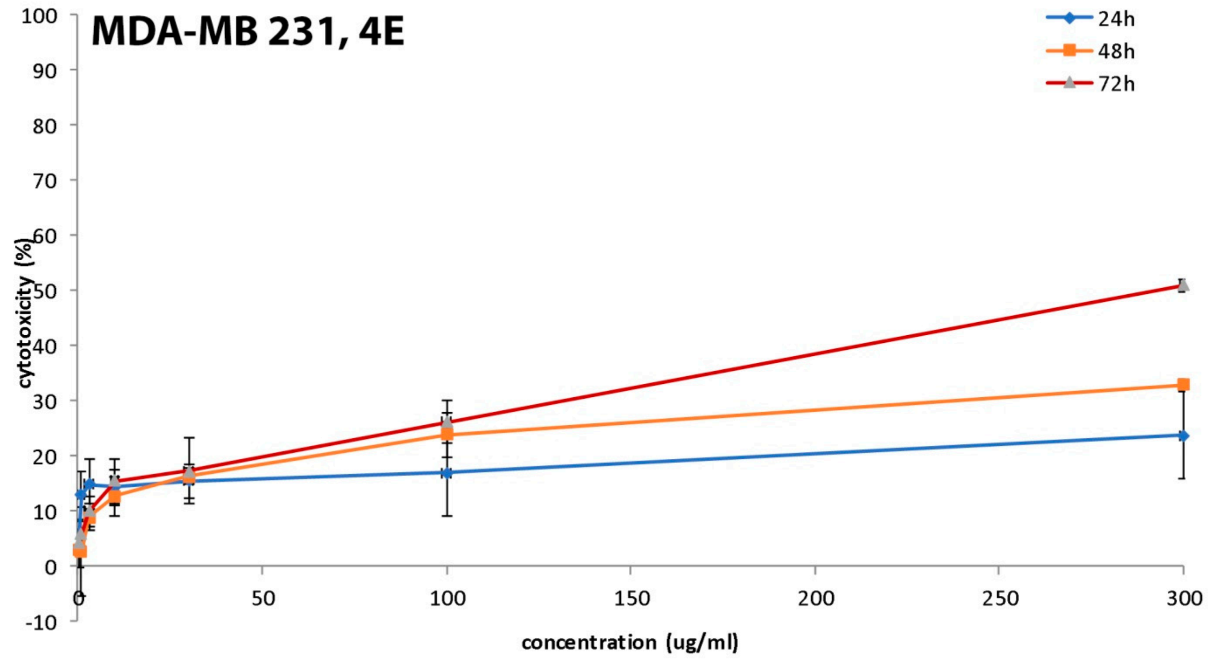

Figure S2(q)

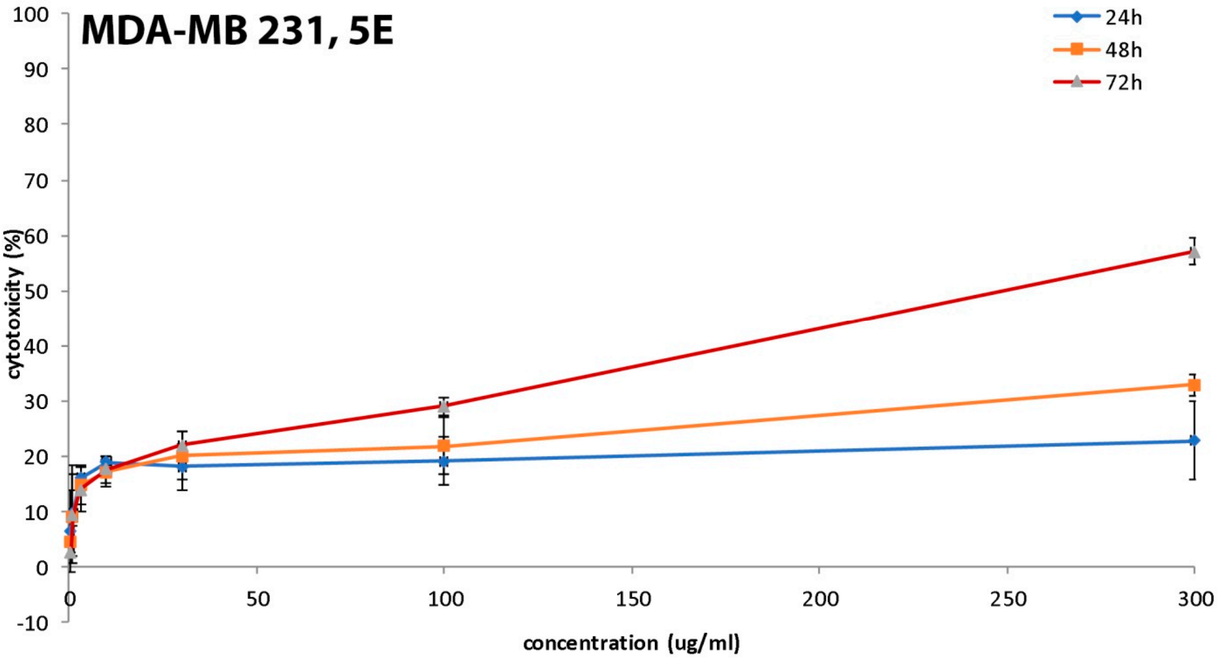

Figure S2(r)

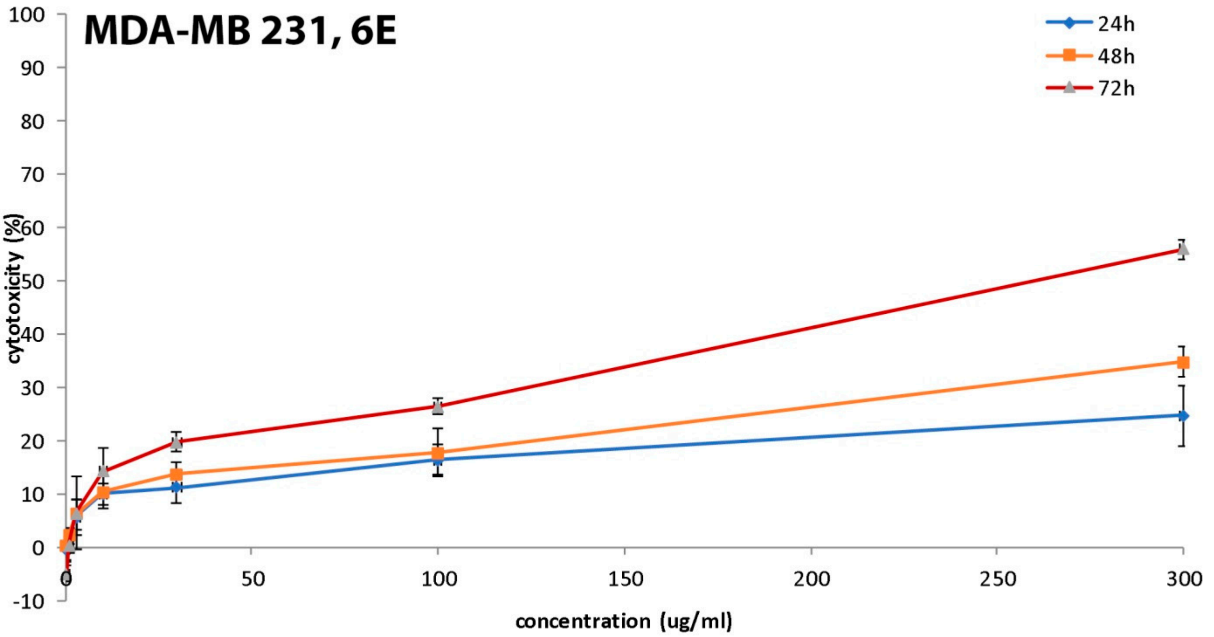

Figure S2(s)

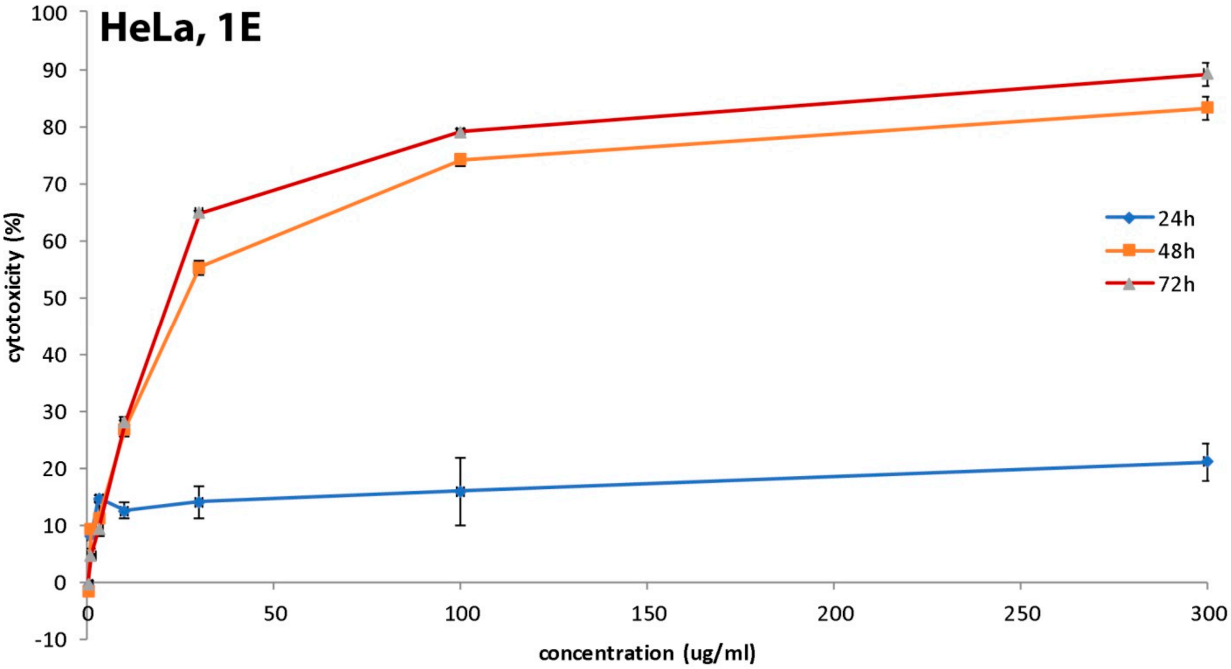

Figure S2(t)

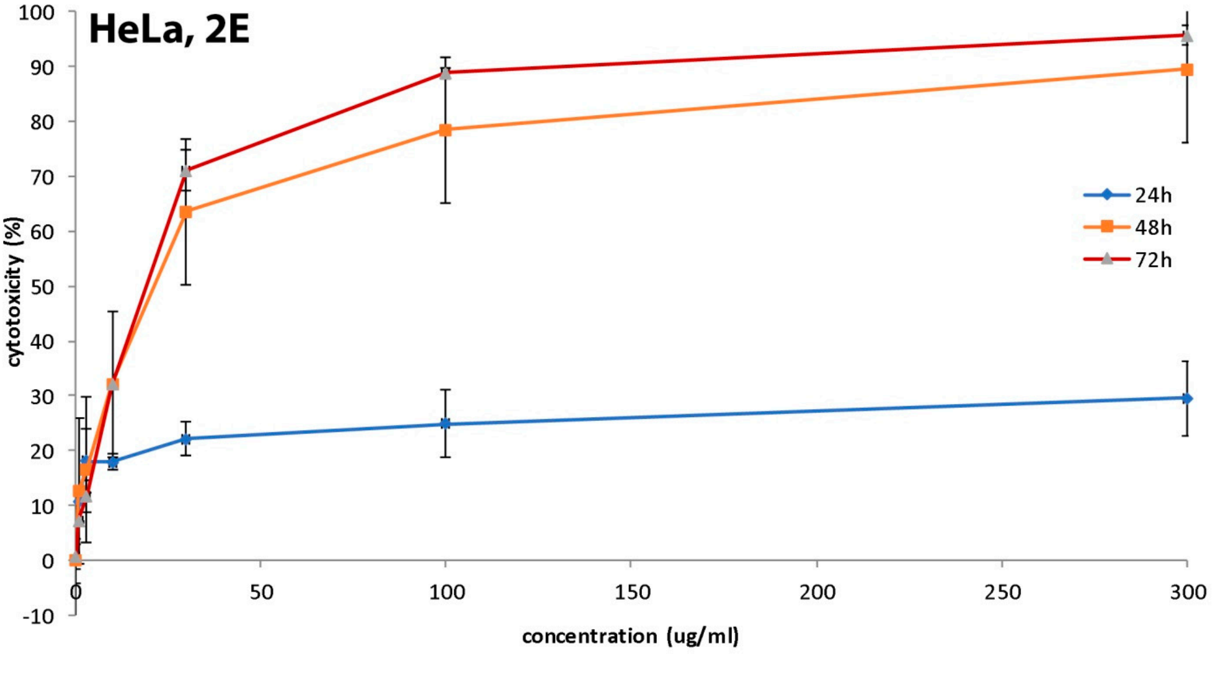

Figure S2(u)

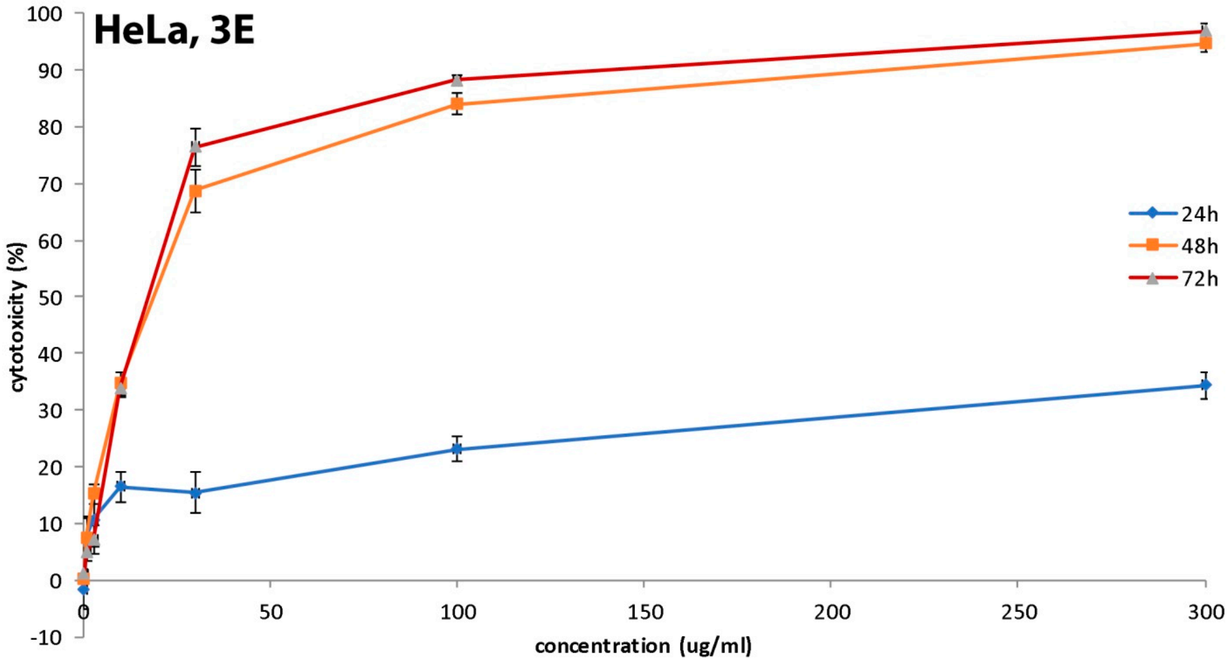

Figure S2(v)

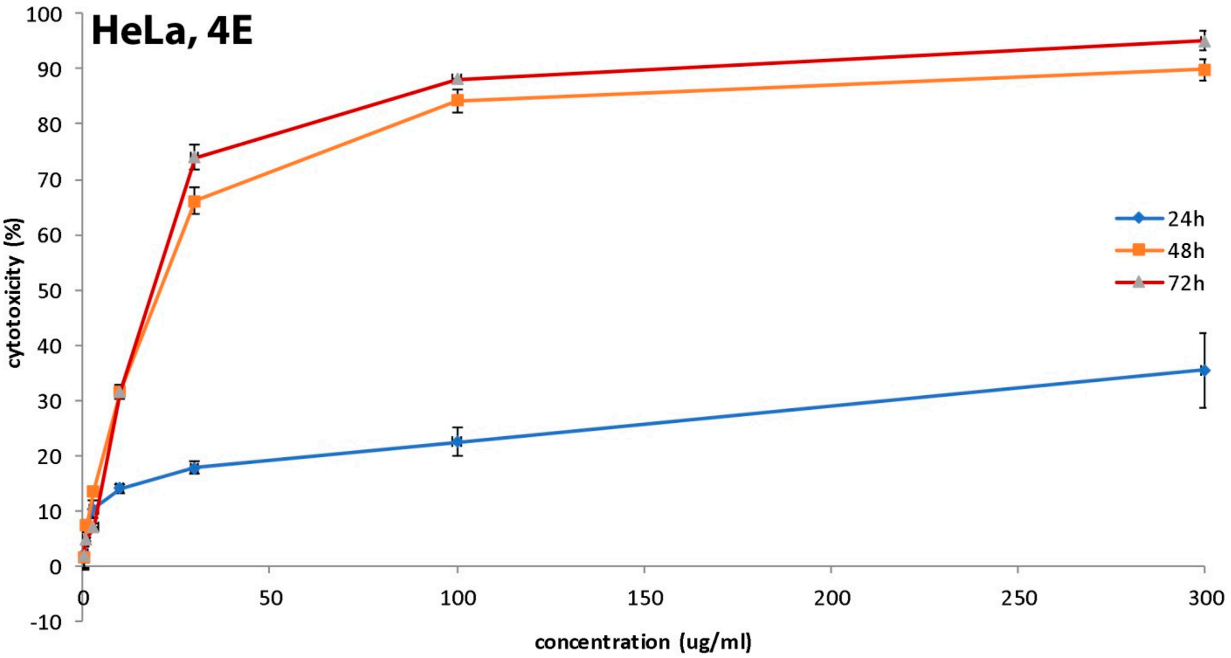

Figure S2(w)

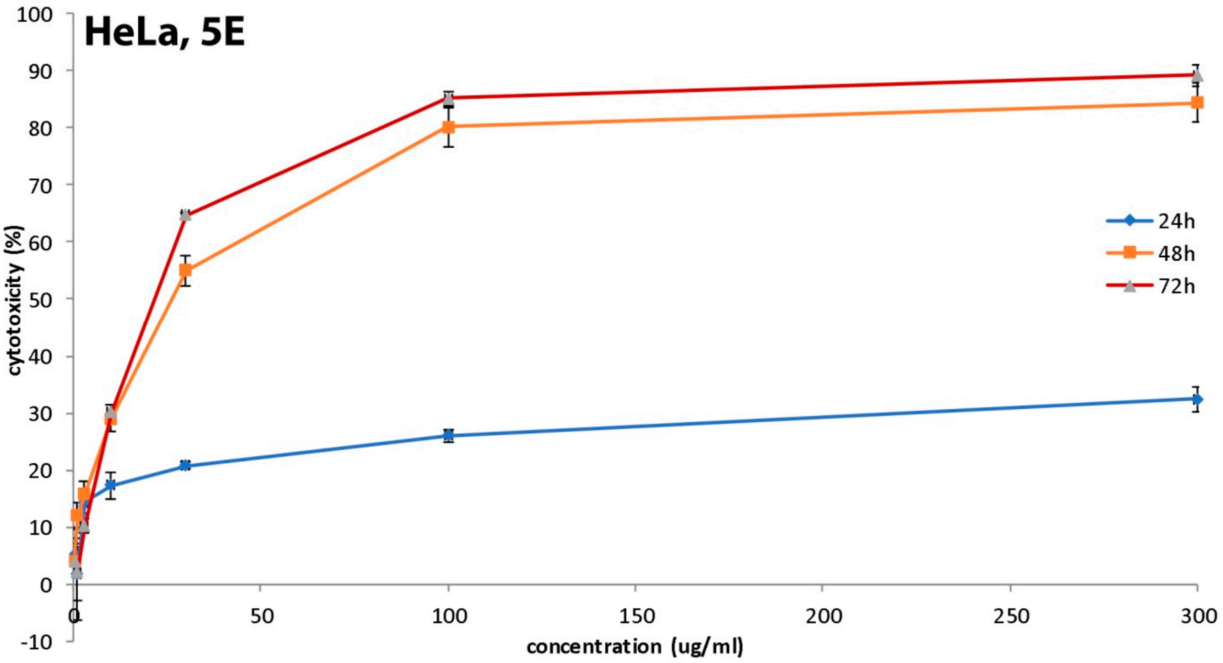

Figure S2(x)

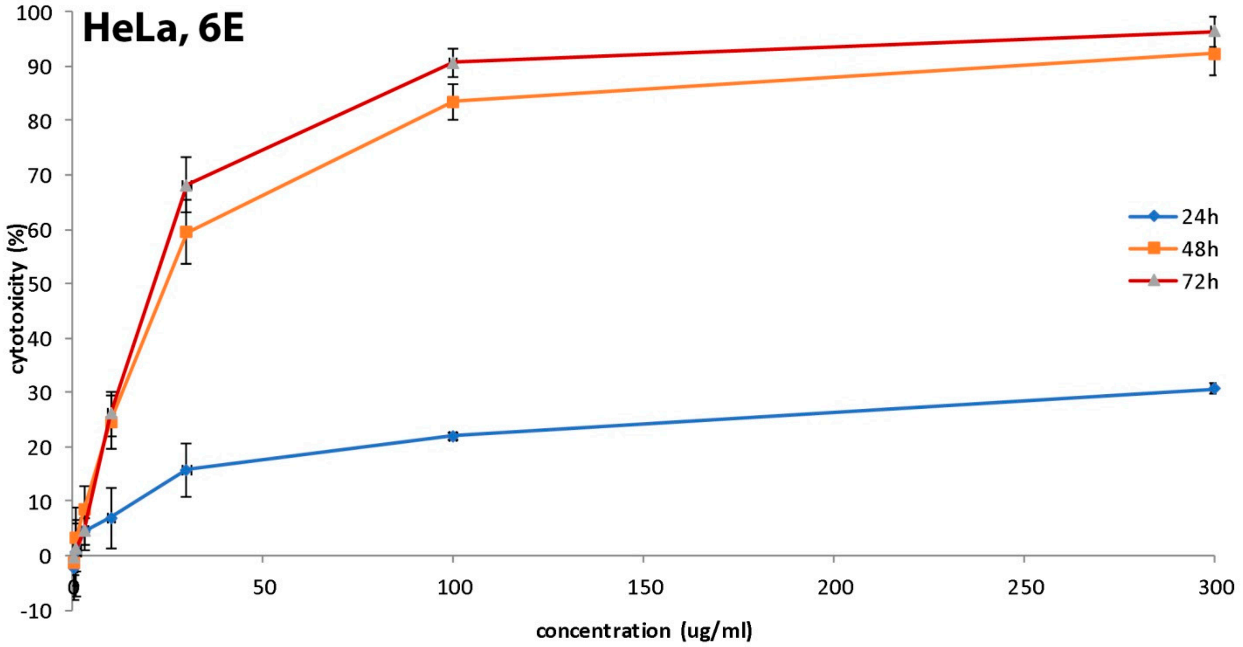

Table S1. Component loadings and score coefficients for constituents of *Hyssopus officinalis* essential oils. Significant characters of separation (factor loadings higher than ± 0.7) were marked bold.

**Variable**	**Component loadings**				**Component score coefficients (eigenvectors)**			
	**PC 1**	**PC 2**	**PC 3**	**PC 4**	**PC 1**	**PC 2**	**PC 3**	**PC 4**
α-Thujene	0.472	0.146	-0.128	**0.858**	0.150	0.061	-0.081	0.607
α-Pinene	**-0.707**	0.700	0.093	-0.008	-0.224	0.292	0.059	-0.006
Sabinene	**-0.863**	-0.019	0.473	0.060	-0.274	-0.008	0.299	0.042
β-Pinene	**0.821**	0.285	0.476	-0.134	0.260	0.119	0.301	-0.095
β-Myrcene	**-0.798**	-0.271	0.412	-0.021	-0.253	-0.113	0.260	-0.015
p-Cymene	-0.562	**0.808**	-0.145	0.016	-0.178	0.337	-0.091	0.012
Limonene	**0.902**	-0.239	-0.205	0.292	0.286	-0.100	-0.130	0.206
1,8-Cineole	**-0.905**	-0.324	-0.237	0.095	-0.287	-0.135	-0.150	0.067
Z-*β*-Ocimene	**-0.866**	-0.301	-0.164	0.310	-0.275	-0.126	-0.104	0.219
E-*β*-Ocimene	-0.645	-0.490	0.552	0.050	-0.205	-0.204	0.349	0.036
γ-Terpinene	**-0.745**	0.610	0.190	0.097	-0.236	0.254	0.120	0.069
trans-Pinocarveol	-0.274	**0.915**	-0.091	-0.266	-0.087	0.381	-0.057	-0.188
trans-Pinocamphone	**0.851**	0.166	0.163	0.376	0.270	0.069	0.103	0.266
Pinocarvone	0.550	0.367	0.387	-0.609	0.174	0.153	0.244	-0.430
cis-Pinocamphone	0.813	0.204	0.403	0.011	0.258	0.085	0.254	0.008
Myrtenal	-0.311	**0.939**	-0.147	-0.015	-0.099	0.391	-0.093	-0.011
Myrtenyl acetate	-0.427	**0.893**	-0.098	0.073	-0.135	0.372	-0.062	0.052
β-Bourbonene	-0.143	-0.348	**-0.812**	-0.444	-0.045	-0.145	-0.513	-0.314
Methyl eugenol	**0.775**	-0.214	0.091	-0.411	0.246	-0.089	0.057	-0.290
E-*β*-Caryophyllene	-0.645	-0.490	0.552	0.050	-0.205	-0.204	0.349	0.036
Germacrene D	-0.642	-0.667	-0.149	-0.290	-0.204	-0.278	-0.094	-0.205
